# Exploring the role of antioxidants in sepsis-associated oxidative stress: a comprehensive review

**DOI:** 10.3389/fcimb.2024.1348713

**Published:** 2024-03-06

**Authors:** Dipak Kumar Sahoo, David Wong, Anil Patani, Biswaranjan Paital, Virendra Kumar Yadav, Ashish Patel, Albert E. Jergens

**Affiliations:** ^1^ Department of Veterinary Clinical Sciences, College of Veterinary Medicine, Iowa State University, Ames, IA, United States; ^2^ Department of Biotechnology, Smt. S. S. Patel Nootan Science and Commerce College, Sankalchand Patel University, Gujarat, India; ^3^ Redox Regulation Laboratory, Department of Zoology, College of Basic Science and Humanities, Odisha University of Agriculture and Technology, Bhubaneswar, India; ^4^ Department of Life Sciences, Hemchandracharya North Gujarat University, Gujarat, India

**Keywords:** sepsis, antioxidants, inflammation, immunosuppression, organ dysfunction, antimicrobial, ROS, oxidative stress

## Abstract

Sepsis is a potentially fatal condition characterized by organ dysfunction caused by an imbalanced immune response to infection. Although an increased inflammatory response significantly contributes to the pathogenesis of sepsis, several molecular mechanisms underlying the progression of sepsis are associated with increased cellular reactive oxygen species (ROS) generation and exhausted antioxidant pathways. This review article provides a comprehensive overview of the involvement of ROS in the pathophysiology of sepsis and the potential application of antioxidants with antimicrobial properties as an adjunct to primary therapies (fluid and antibiotic therapies) against sepsis. This article delves into the advantages and disadvantages associated with the utilization of antioxidants in the therapeutic approach to sepsis, which has been explored in a variety of animal models and clinical trials. While the application of antioxidants has been suggested as a potential therapy to suppress the immune response in cases where an intensified inflammatory reaction occurs, the use of multiple antioxidant agents can be beneficial as they can act additively or synergistically on different pathways, thereby enhancing the antioxidant defense. Furthermore, the utilization of immunoadjuvant therapy, specifically in septic patients displaying immunosuppressive tendencies, represents a promising advancement in sepsis therapy.

## 1 Introduction

Sepsis is a worldwide health concern characterized by life-threatening organ dysfunction triggered by a dysregulated host response to an infection (What is Sepsis? | Sepsis | CDC, n.d.; Seymour et al., 2016; Singer et al., 2016; Reinhart et al., 2017; Rhee et al., 2019; Dong et al., 2020; Dupuis et al., 2020). Recent data from the Centers for Disease Control and Prevention suggest that approximately 1.7 million adults in the United States are affected by sepsis yearly, with an estimated 350,000 deaths attributable to the condition (What is Sepsis? | Sepsis | CDC, n.d). Furthermore, studies have found that between 30-50% of hospital deaths are caused by sepsis (What is Sepsis? | Sepsis | CDC, n.d.; Rhee et al., 2019). The prevailing circumstances are notably exacerbated in low- and middle-income countries, where the occurrence and death rate of sepsis are markedly greater, and also in the locations that have the least resources to prevent, detect, or treat sepsis (**Table 1**) (Sepsis, n.d.; Rudd et al., 2020). Regardless of a country’s economic and healthcare status, sepsis remains a persistent and universal medical concern, as evidenced by the World Health Organization (WHO) designating sepsis as a global healthcare issue (https://www.who.int/news-room/fact-sheets/detail/sepsis) (Sepsis, n.d). It is crucial to evaluate and enforce stronger infection-prevention measures in regions with the highest sepsis rates, especially among vulnerable populations like neonates (Rudd et al., 2020). Numerous therapeutic improvement projects to treat sepsis have been launched in hospitals worldwide in response to the high incidence of sepsis and the belief that most sepsis-associated deaths are preventable with improved care. However, it is unlikely that better hospital-based care can prevent the majority of sepsis-associated deaths. Additional advancements in the prevention and management of underlying conditions may be required to significantly decrease mortality rates associated with sepsis (Rhee et al., 2019). Though sepsis is a multifaceted physiological response to infection that affects the entire organism causing organ dysfunction, septic shock is a “subset of sepsis characterized by severe circulatory, cellular, and metabolic abnormalities that pose a higher risk of mortality compared to sepsis alone” (Tanaka et al., 2000; Singer et al., 2016). The most severe cases tend to exhibit elevated levels of plasma glucose (Freire Jorge et al., 2017), triglycerides (Lee et al., 2015), and lactate (Nichol et al., 2010; Casserly et al., 2015; Shankar-Hari et al., 2016), while hypoglycemia in combination with hyperlactatemia has been associated with increased incidences of renal and liver dysfunction and mortality (Freire Jorge et al., 2017). Multiple processes, including hypoxia and oxidative stress (OS), contribute to sepsis, characterized by systemic inflammation in response to bacterial infection.

**Table 1:** Sepsis-related deaths in different countries demonstrating its severity.

	Name of the country/region	Cases of sepsis incidents	Sepsis-related deaths	Deaths from sepsis incidents (%)	Sepsis-related deaths due to infection	Deaths caused by sepsis linked to non-communicable diseases	Sepsis related deaths due to injury
Countries with the highest prevalence of sepsis cases	India	11,341,313	2,942,916	25.95	1,648,322	1,172,987	155,628
	Nigeria	5,333,767	753,357	14.12	594,159	148,604	15,483
	China	2,931,821	709,315	24.19	196,016	484,305	38,835
	Pakistan	2,144,423	443,007	20.66	202,008	226,690	20,343
	Indonesia	1,635,563	422,140	25.81	200,420	213,583	14,044
	Democratic Republic of the Congo	1,361,764	310,088	22.77	216,221	84,266	11,651
	Bangladesh	1,147,722	213,628	18.61	97,199	112,200	6,974
	USA	1,083,007	189,623	17.51	77,456	109,865	6,912
	Ethiopia	1,018,392	268,383	26.35	176,253	84,011	9,724
	Brazil	879,132	217,618	24.75	104,269	108,243	10,059
	Philippines	763,194	161,259	21.13	92,732	65,089	4,880
	Kenya	594,293	117,126	19.71	83,288	31,098	3,549
	Tanzania	586,334	149,449	25.49	97,257	49,002	4,370
	Russia	573,487	141,660	24.7	45,890	91,020	7,870
	Niger	568,605	101,711	17.89	80,555	19,318	2,507
	Mali	568,495	92,171	16.21	64,893	25,202	2,823
	Mexico	483,584	105,955	21.91	35,685	66,185	5,660
	Burkina Faso	470,981	95,694	20.32	70,458	23,384	2,878
	Japan	470,031	95,616	20.34	53,361	41,743	1,891
	Egypt	467,600	80,662	17.25	39,487	37,688	5,226
Regions worldwide with the highest incidence of sepsis cases	Sub-Saharan Africa	16,681,190	3,492,058	20.93	2,498,546	905,957	114,375
	South Asia	14,830,172	3,655,232	24.65	1,975,574	1,537,091	185,971
	Southeast Asia, east Asia, and Oceania	7,137,538	1,781,344	24.96	721,104	1,001,984	82,690
	North Africa and Middle East	2,754,140	497,164	18.05	189,894	240,288	77,467
	Latin America and Caribbean	2,479,512	553,044	22.30	244,159	292,182	27,452
	Central Europe, eastern Europe, and central Asia	1,539,623	364,724	23.69	122,996	233,937	16,924

Globally, there have been a documented total of 48.9 million identified cases of sepsis, with around 11 million recorded deaths representing approximately 20% of the total global mortality rate. The data presented here is from 2017 and has been adapted from a previous report by Rudd et al. (2020).

Despite the advancement of our knowledge of sepsis and improved therapeutic modalities, septic shock remains a significant contributor to mortality in intensive care units (ICUs) (Basodan et al., 2022). Though extensive therapeutic studies have been conducted on animals, the results have yet to be successfully translated into human therapeutic practice. There are obvious distinctions between animal models of disease and what is seen in human patients, and it is well-acknowledged that no “one-animal model” can recreate the complex and variable clinical presentations of sepsis syndrome (Nandi et al., 2020). Nevertheless, these studies possess scientific significance in comprehending the fundamental pathophysiology and verifying innovative therapeutic and diagnostic methods, provided that a thorough assessment of the potential risks and benefits has been undertaken and the data is interpreted realistically.

Sepsis is characterized by an exaggerated and imbalanced immune response triggered by an infection. Typically, the course of an infection entails a regulated inflammatory cascade that transpires via the delicate equilibrium between pro- and anti-inflammatory molecules (Esposito et al., 2017). In instances where sepsis remains unregulated, a series of subsequent occurrences may transpire, ultimately leading to the development of septic shock and multi-organ failure (Chiu and Legrand, 2021). Many pathophysiological pathways, including sepsis, inflammation, and organ disorders, have been linked to OS (Abelli et al., 2022). The current review article aims to present a comprehensive overview of the cellular metabolism of reactive oxygen species (ROS) and its involvement in pathophysiological processes, particularly in sepsis. This review article also offers a thorough overview of the potential use of antioxidants specifically with antimicrobial properties, as a supplementary treatment for sepsis, in addition to primary therapies such as fluid and antibiotic therapies. Additionally, the available antioxidants and the potential reasons for their effectiveness or lack thereof in mitigating diseases caused by OS are discussed, along with recent advancements in mitochondria-targeted antioxidants and their potential implications, as well as multi-antioxidant therapy and immunoadjuvant therapy.

### 1.1 Different sepsis types and their mechanisms

Myeloid immune cells, including monocytes, macrophages, and dendritic cells, play a crucial role in the initial response to infections. A variety of innate immune receptors known as pattern recognition receptors (PRRs) identify conserved structural motifs found in microbes and endogenous stress signals known as microbe-associated molecular patterns (MAMPs), pathogen-associated molecular patterns (PAMPs) or damage-associated molecular patterns (DAMPs). The primary families of PRRs consist of toll-like receptors (TLRs), nucleotide oligomerization domain (NOD)-like receptors (Nucleotide-binding oligomerization domain, Leucine-rich Repeat and Pyrin domain containing, also abbreviated as NALP), C-type lectin-like receptors (CLRs), retinoic acid-inducible gene-I (RIG-I)-like receptors (RLRs) and DNA-sensing molecules. After detecting their specific ligands, these receptors trigger innate immune responses to provide immediate protection or coordinate the activation of adaptive immunity (Patin et al., 2019; Yuki and Koutsogiannaki, 2021).

### 1.2 Bacterial sepsis

**Figure 1:**
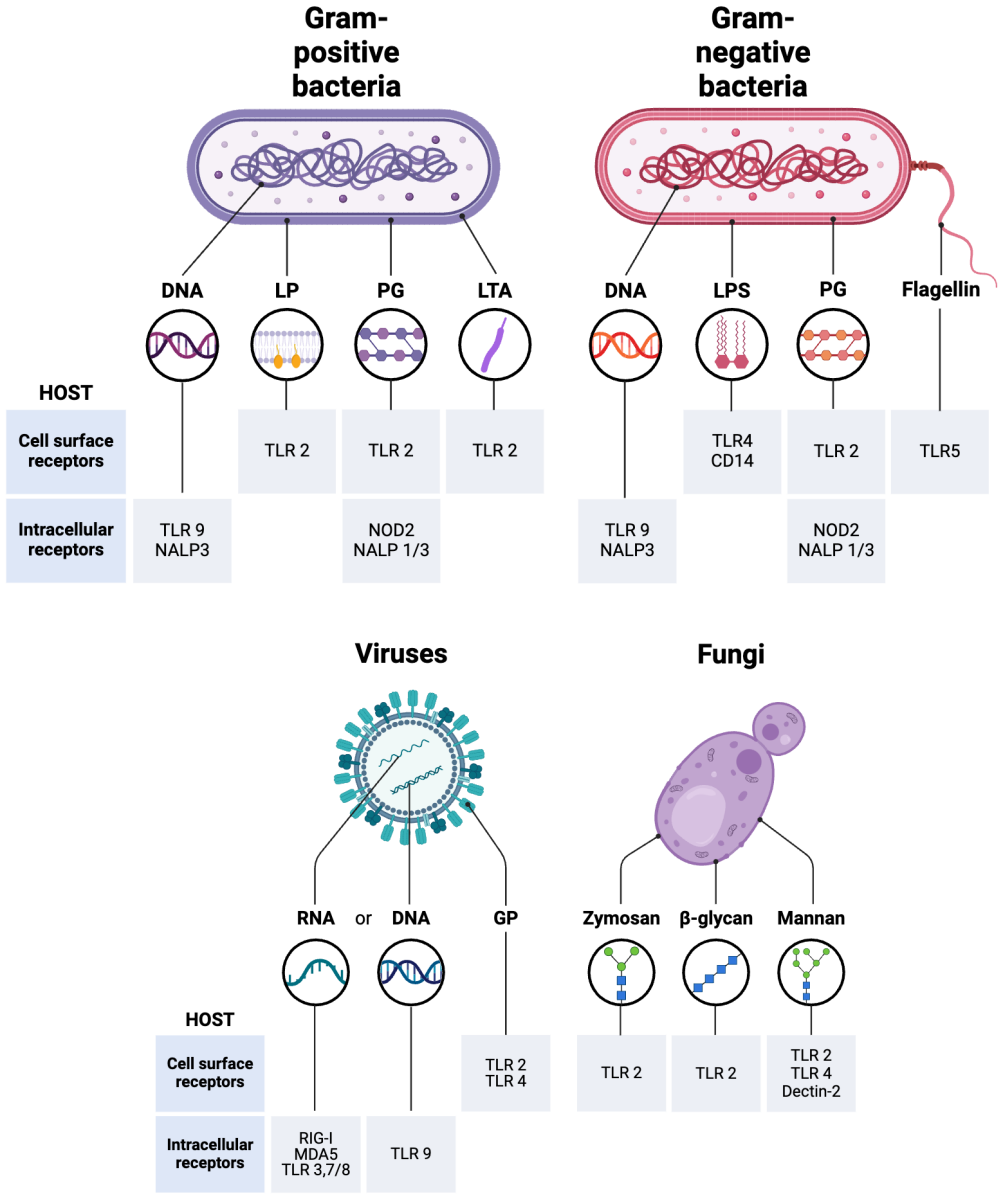
Recognition of Pathogen-Associated Molecular Patterns (PAMPs) that mediate different types of sepsis. LPS, Lipopolysaccharide; TLR, Toll-like receptors; PG, Peptidoglycan; LP, Lipoprotein; LTA, Lipoteichoic acid; GP, Envelope glycoprotein; CD14, cluster of differentiation 14; RIG-I, Retinoic acid-inducible gene-I; NOD-like receptors, Nucleotide oligomerization domain; (NALP, Nucleotide-binding oligomerization domain, Leucine-rich Repeat and Pyrin domain containing; MDA5, Melanoma differentiation-associated protein 5. The figure was generated using BioRender (www.biorender.com; accessed on 7th Feb 2024).

Bacterial pathogens are widely recognized as the predominant etiological agents responsible for the development of sepsis, with 62.2% of sepsis cases with positive blood cultures exhibiting the presence of Gram-negative bacteria (19.9% cases with *Pseudomonas* species, 16% with *Escherichia coli*, 12.7% with *Klebsiella* species, 8.8% cases with *Acinetobacter* species and *Enterobacter* 7%) and 46.8% of patients with Gram-positive bacterial infections (*Staphylococcus aureus* 20.5%, methicillin-resistant *S. aureus* 10.2%, *Enterococcus*10.9%, *S. epidermidis* 10.8%) (Mayr et al., 2014). Irrespective of the specific bacterial strain, it is noteworthy that various components of the overarching mechanism underlying bacterial sepsis exhibit a high degree of conservation. Bacterial surface toxins like lipopolysaccharide (LPS) or bacterial-secreted PAMPs elicit activation of TLRs and other cell-surface receptors present on host cells (Dolin et al., 2019). The cell walls of Gram-positive bacteria are primarily composed of peptidoglycan, lipoproteins and glycolipid lipoteichoic acid. While peptidoglycan is detected by TLR2 and NOD-containing protein-1 (NOD-1) and -2 (NOD-2), TLR2 recognizes lipoteichoic acid and lipoproteins and TLR9 has the ability to detect unmethylated CpG motifs in bacterial DNA (**Figure 1**). The cell walls of Gram-negative bacteria have peptidoglycan, lipopolysaccharide (LPS), phospholipids, and proteins. While LPS is detected by the MD-2/TLR4 complex, TLR5 recognizes flagellin, a significant constituent of flagella, primarily present in Gram-negative bacteria (**Figure 1**) (Yuki and Koutsogiannaki, 2021). Subsequently, intracellular signaling triggers the initiation of pro-inflammatory cascades, thereby facilitating the recruitment of additional inflammatory cells. This cascade, along with endothelial dysfunction, coagulopathy, and cellular and cardiovascular dysfunction, rather than the mere presence of bacteremia, is responsible for advancing multiorgan failure in sepsis (Evans, 2018).

### 1.3 Viral sepsis

The incidence of viral sepsis is quite low, with the highest susceptibility to viral-induced sepsis involving the pediatric and geriatric populations (Mayr et al., 2014). While TLR2 and TLR4 are present on the cell surface and detect viral proteins, intracellular TLRs such as TLR3, TLR7, TLR8, and TLR9 are present in endosomes and detect viral nucleic acids (**Figure 1**). The intracellular TLR3, TLR7/TLR8, and TLR9 detect viral dsRNA, ssRNA, and unmethylated CpG DNA, respectively (Lester and Li, 2014). Proteins known as RIG-I-like receptors, such as RIG-I (ssRNA sensor) and MDA-5 (dsRNA sensor), play a crucial role in detecting viral RNAs generated within a cell (**Figure 1**) (Yuki and Koutsogiannaki, 2021). Viral sepsis in individuals is primarily manifested because of impaired interferon (IFN) signaling pathways facilitated by viral agents. The initiation of antiviral signaling is mediated by the activation of type I α/β IFN, in addition to IFN-γ, a type II IFN that is accountable for the induction of chemokine and pro-inflammatory cytokine signaling (Kelly-Scumpia et al., 2010). The aforementioned proteins subsequently elicit pro-inflammatory responses primarily via the phosphorylation of proteins, which in turn trigger the synthesis of additional IFN (Baccala et al., 2014). While cytokine-dependent signaling is also initiated, it is noteworthy that the IFN system serves as the principal mechanism employed by the host to combat viral infections.

### 1.4 Fungal sepsis

Even though fungi are a component of the normal flora in numerous body regions (in the gut, on the skin, in the mouth, and other mucosal surfaces), fungal sepsis exhibits a rapid growth rate and is frequently fatal in nature, exhibiting a mortality range of 40% to 60% among patients with invasive candidiasis or candidemia (Upperman et al., 2003; Delaloye and Calandra, 2014; Dolin et al., 2019). *Candida* species account for an estimated 17% of sepsis cases, while an additional 1.4% can be attributed to *Aspergillus*. Fungal sepsis commonly arises from reactions to specific toxins and byproducts produced by fungi. For example, gliotoxin, a fungal metabolite, has the potential to induce sepsis *in vivo* by causing damage to gut tissue as it triggers enterocyte apoptosis (Upperman et al., 2003). Fungal sepsis is predominantly sustained via signal transduction cascades, specifically involving the activation of IL-17 (Netea et al., 2015). The body’s ability to sense fungal antigens also involves a wide variety of cell-surface receptors. The PRRs detect a range of fungal cell wall components, including mannans, mannoproteins, β-glucans, chitin, and also fungal-derived RNA and unmethylated DNA. After ligand binding, PRRs play a crucial role in shaping immune responses. After binding to ligands, PRRs initiate different signaling pathways that lead to the internalization of fungi through phagocytosis, the production of cytokines, and/or RNS and ROS. It has been demonstrated that TLR2 is recruited to phagosomes containing zymosan and β-glucans (Patin et al., 2019) while TLR2, TLR4, and dectin‐2 play significant roles in detecting fungal mannans (**Figure 1**) (Hall and Gow, 2013; Onyishi et al., 2023). The administration of exogenous IL-10, a cytokine known for its immunosuppressive properties, has demonstrated detrimental effects in experimental models of fungal infection (Clemons et al., 2000). The concentration of IL-10 has been observed to be elevated in the plasma of individuals diagnosed with septic shock. Studies have demonstrated a correlation between IL-10 blood levels, the severity of inflammation, and the occurrence of organ failure in septic shock (Friedman et al., 1997).

## 2 Sepsis and oxidative stress

**Figure 2:**
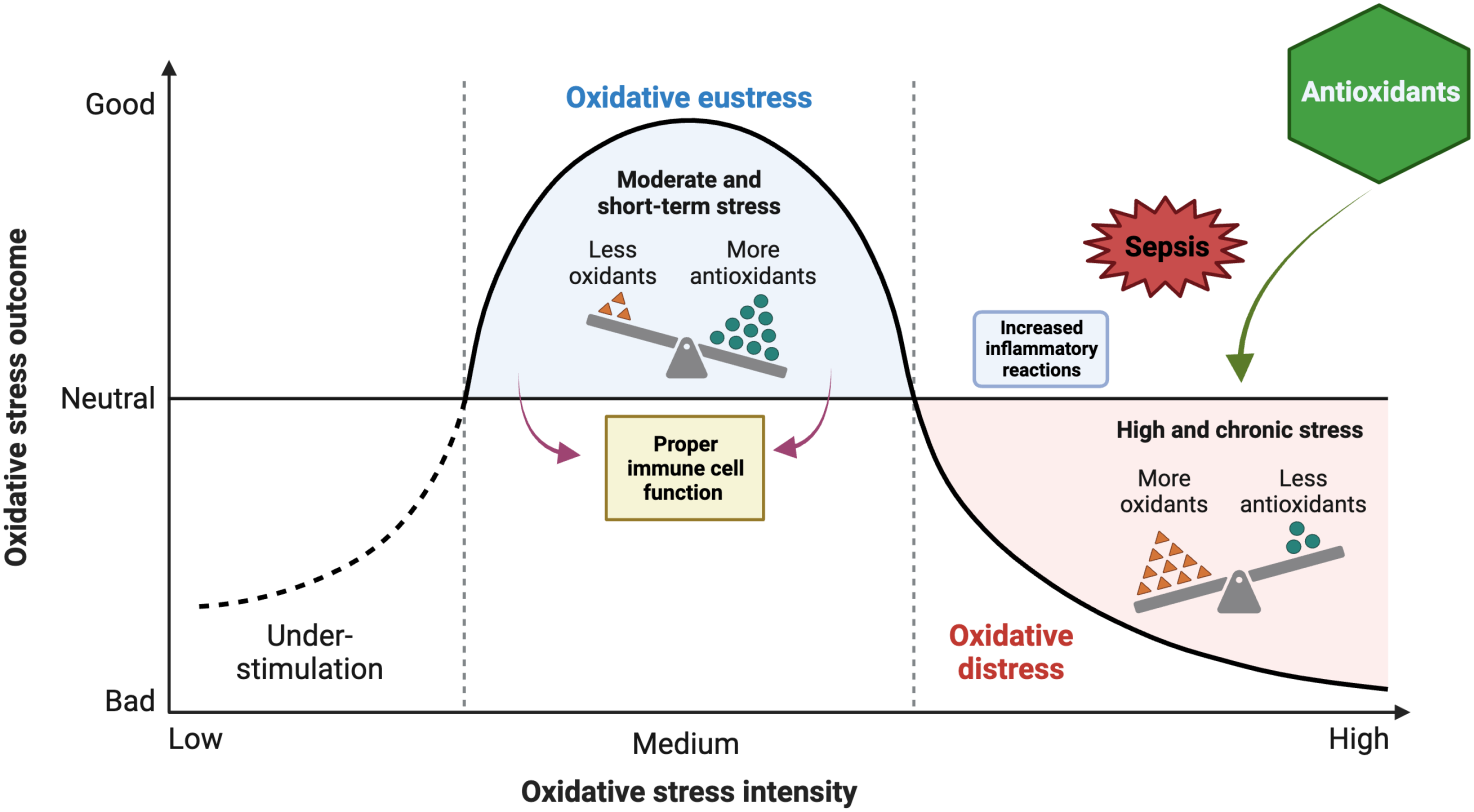
Impacts of oxidant and antioxidant imbalance on oxidative stress (OS) outcome during sepsis and potential protective role of antioxidants. The figure was generated using BioRender (www.biorender.com; accessed on 1st Nov 2023).

While OS refers to an imbalance between oxidants and antioxidants, favoring the oxidants, this imbalance consequently disrupts redox signaling and regulation and can potentially cause harm at the molecular level. While oxidative eustress refers to a low-level (physiological) OS that plays a role in redox signaling and regulation, oxidative distress refers to a supraphysiological oxidative challenge that results in impaired redox signaling and/or oxidative damage to biomolecules (Sies, 2019) (**Figure 2**). The significance of ROS in serving as signaling molecules for the facilitation of regular cellular processes and the ability to adapt to cellular stress cannot be ignored. One of these adaptations is the response to infection, both a cause and a consequence of critical illness. The regulation of ROS has the potential to modulate the extent of the inflammatory response.

Sepsis is a complex syndrome with multiple contributing factors. As a life-threatening complication caused by OS and inflammatory reactions, it represents a leading contributor to mortality in critically ill individuals. Several molecular mechanisms underlying the progression of sepsis are associated with the dysregulation between the generation of ROS and its elimination through cellular antioxidant pathways (Abelli et al., 2022). In sepsis, excessive generation of oxygen free radicals contributes to the development of multiple organ failure and mortality (Cowley et al., 1996).

The pathogenesis of sepsis encompasses infection by gram-positive and gram-negative bacteria, fungi, or a combination thereof. Sepsis is characterized by an intensified inflammatory response and in severe cases, hypotension, vasodilatory shock, and reduced oxygen delivery to tissues can occur due to cellular impairment of oxygen utilization. Though ROS and reactive nitrogen species (RNS) are involved in the progression of sepsis, their precise significance and function remain unclear. However, hyperpermeability, hypotension resulting from decreased vascular resistance, and mitochondrial dysfunction in respiration are crucial factors contributing to multiorgan failure and, ultimately, fatality in individuals with sepsis. The involvement of ROS/RNS, namely superoxide (O_2_−), hydroxyl radical (^•^OH), hydrogen peroxide (H_2_O_2_), nitric oxide (NO^•^), and peroxynitrite (ONOO−), has been documented as one of the underlying mechanisms of these effects. The impairment of mitochondrial function is linked with depletion of the inner membrane and the hindrance of the electron transfer chain and FoF1-adenosine triphosphate-synthase within the mitochondria, leading to decreased cellular energy production. Furthermore, the excessive generation of NO^•^ resulting from inducible nitric oxide synthase (iNOS) activity is linked to detrimental consequences such as widespread vasodilation and reduced sensitivity to exogenously administered vasopressors (Prauchner, 2017). Sepsis is characterized by the induction of iNOS production in diverse cells due to the action of inflammatory mediators and cytokines.

Several studies on sepsis have demonstrated the presence of oxidative imbalance and increased OS due to a combination of factors, including hypotension, microvascular thrombosis, mitochondrial damage from OS, and other mechanisms, such as inflammation (Angus and van der Poll, 2013). An investigation by Takeda et al. observed that septic patients exhibited elevated levels of thiobarbituric acid reactive substance (TBARS), indicating a rise in lipid peroxidation (LPx) (Takeda et al., 1984). A reduction in the levels of antioxidants was also observed (Goode et al., 1995). According to Cowley et al., individuals who survived sepsis exhibited higher levels of antioxidant potential than those who did not (Cowley et al., 1996). Additionally, the antioxidant potential was observed to increase rapidly and reach normal or supranormal levels. Two additional prospective observational studies have demonstrated a correlation between total antioxidant capacity and the Acute Physiology and Chronic Health Evaluation II (APACHE II) score (Chuang et al., 2006), as well as a correlation between antioxidant deficiency and mortality (Karapetsa et al., 2013). Survival was associated with stronger catalase (CAT) activity, lower protein carbonyl (PC) levels, and stable glutathione (GSH) levels in erythrocytes. A study has reported a significant decrease in plasma vitamin C levels among individuals diagnosed with multiorgan failure (Borrelli et al., 1996). The deleterious effects of increased levels of oxidants in sepsis involve the alteration of proteins, lipids, and nucleic acids, which subsequently results in cellular damage and impaired endothelial function. Furthermore, the deterioration of glycocalyx and intercellular junctions among endothelial cells results in increased vascular permeability, a fundamental aspect in the progression of sepsis (Rubio-Gayosso et al., 2006).

Activation of nuclear factor kappa B and treatment with lipopolysaccharide (LPS) increase inducible nitric oxide synthase (iNOS) activity, leading to increased levels of nitric oxide (NO) that can react with superoxide anions to generate ONOO− (Brown, 2001). A similar correlation between OS and mortality in sepsis has been observed between the SOD: CAT ratio. Manganese-containing superoxide dismutase (Mn-SOD) typically converts superoxide to H_2_O_2_; subsequently, CAT converts H_2_O_2_ to water. However, the quantities of these enzymes vary under conditions of high OS, such as sepsis, which may lead to an accumulation of harmful ROS (Andrades et al., 2005).

Clinical oxidative indicators that can detect the byproducts of ROS damage to membrane lipids, proteins, and nuclear components include measurement of malondialdehyde (MDA), F2-isoprostanes, and 8-hydroxy-2’-deoxyguanosine (Sahoo and Chainy, 2023). Endogenous antioxidant enzymes, including CAT, SOD, and glutathione peroxidases (GPx), also serve as important biomarkers of OS. Another indicator is the ratio of reduced to oxidized thiols. Electrochemical measuring of derived reactive oxygen metabolites (dROM) (d-ROM, an index of OS) or biological antioxidant potential (BAP) or the redox balance (d-ROM/BAP ratio) is now possible because of recent technological advancements (Cortese-Krott et al., 2017; Araki et al., 2018; Miripour et al., 2022). Point-of-care (POC) systems have made it possible to quickly assess OS levels with a minimal blood sample (Cortese-Krott et al., 2017; Araki et al., 2018; Miripour et al., 2022).

Using a porcine model examining the alterations in the plasma proteome associated with sepsis revealed that there were changes in the plasma levels of 36 proteins, with 30 proteins being upregulated and 6 proteins being downregulated. These proteins represent a total of 27 unique proteins, as determined by differential proteomics and include CD14, haptoglobin and haemopexin (Thongboonkerd et al., 2009). CD14 is linked to acute-phase reaction proteins and OS pathways, whereas hemopexin acts as both an anti-inflammatory agent and oxidative scavenger.

The receptor for bacterial lipopolysaccharide (LPS) is CD14, which works together with TLR4 and MD-2 to activate the innate immune response (**Figure 1**) (Frantz et al., 2007; Sahoo et al., 2022). Haptoglobin is an acute-phase reaction protein that binds to hemoglobin, preventing renal iron loss and oxidative damage caused by free hemoglobin (Lim et al., 2000; Van Campenhout et al., 2006). Hemopexin is responsible for binding to heme and facilitating its transportation to the liver, where it undergoes breakdown and enables the recovery of iron. Elevated levels of hemopexin, an anti-inflammatory molecule and oxidative scavenger, were observed during the early stages of sepsis, as heme is known to be highly toxic to cells due to its pro-inflammatory and oxidative effects (Van Campenhout et al., 2006; Thongboonkerd et al., 2009). The High-mobility group box 1 (HMGB1) protein, a nuclear chromatin-binding protein associated with damage-associated molecular patterns (DAMPs), also plays a crucial role in OS regulation and subsequent cellular processes such as apoptosis (Tang et al., 2011). Additionally, HMGB1 plays a crucial role in mediating delayed endotoxin lethality and is indispensable for the complete manifestation of inflammation in animal models of endotoxemia, sepsis, and arthritis. The efficacy of administering HMGB1 blockade after a delay of up to 24 hours following the initiation of experimental sepsis presents a distinctive temporal window that affords potential for rescuing individuals from fatal septic conditions (Andersson and Tracey, 2003).

As organoids offer numerous advantages over conventional models and have been extensively utilized in both fundamental and clinical research (Kopper et al., 2021; Minkler et al., 2021; Bedos et al., 2022; Gabriel et al., 2022a; Gabriel et al., 2022b; Moshksayan et al., 2023; Sahoo et al., 2023b; Gabriel et al., 2024), there are also reports using renal and intestinal organoids in sepsis. The use of organoids pre-treated with LPS serves as an effective means to study the impact of LPS-induced intestinal injury, which closely resembles sepsis, and facilitates the investigation of immune-associated mechanisms as well as the screening of potential therapeutic agents (Huang et al., 2022). Kidney organoids derived from human pluripotent stem cells (hPSCs) were used to investigate the effects of methylprednisolone (MP), a synthetic corticosteroid, on LPS-induced OS and injury. LPS is commonly used to simulate sepsis-associated acute kidney injury (SA-AKI) in both *in vivo* and *in vitro* models (Zhang W. et al., 2021). The results showed that MP partially alleviated the injury by reducing OS (by lowering induced MDA content and myeloperoxidase (MPO) expression and increasing SOD activity) and apoptosis in kidney cells (Zhang W. et al., 2021).

## 3 Sepsis and mitochondria

**Figure 3:**
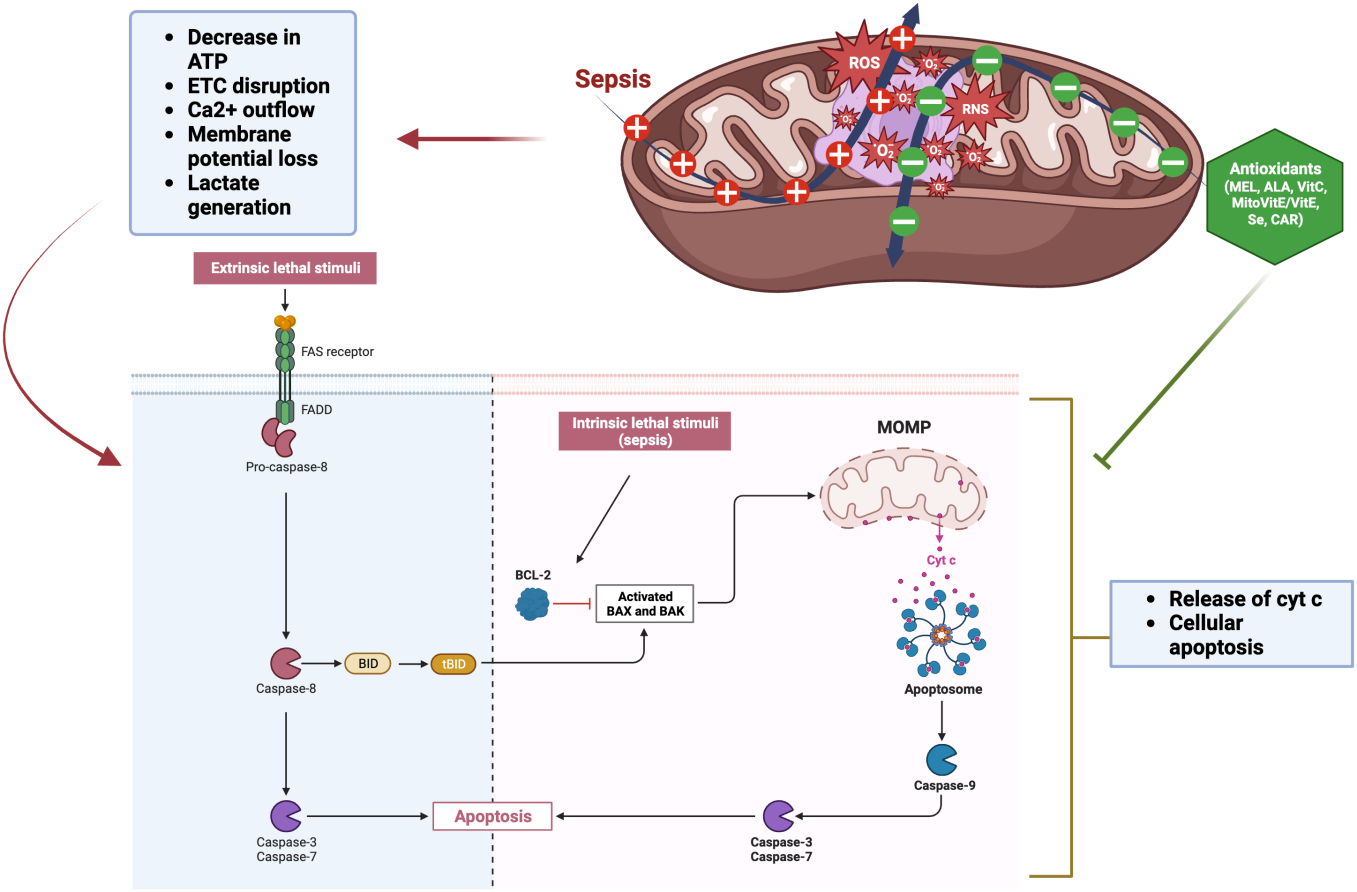
Mitochondrial dysfunction by sepsis and role of antioxidants. The extrinsic pathway is initiated by an extracellular death signal that binds to its receptor. This binding activates caspase-8 through the Fas-associated death domain (FADD). Caspase-8 triggers the activation of apoptotic caspases, specifically caspases -3 and -7. BID, a member of the Bcl-2 family that promotes apoptosis, is activated, and its truncated form (tBID) then triggers the mitochondrial apoptotic pathway. The intrinsic pathway is initiated by endogenous cellular injury, such as ROS generated in sepsis. Pro-apoptotic Bcl-2 proteins (Bax and Bak) play a crucial role in regulating the intrinsic pathway of apoptosis. Following apoptotic signals, these proteins become active and form oligomers at the mitochondrial outer membrane, facilitating its permeabilization. Mitochondrial outer membrane permeabilization (MOMP) facilitates the release of pro-apoptotic proteins, including cytochrome c, from the mitochondria into the cytosol. These proteins then interact with dATP, apoptotic protease activating factor-1 (Apaf-1), and caspase-9 to form the apoptosome, which activates caspase-3 and −7, leading to cell apoptosis. The figure was generated using BioRender (www.biorender.com; accessed on 1^st^ Nov 2023). “+”: Increased ROS generation due to sepsis; “—”: Decreased ROS due to antioxidant treatment; Cyt c, Cytochrome c; Bcl-2, B-cell lymphoma 2; BID, BH3 interacting domain death agonist; BAX, BCL2 associated X; BAK, Bcl-2 antagonist killer 1; MEL, Melatonin; ALA, alpha-Lipoic acid; Vit C, Vitamin C; Vit E, Vitamin E; Se, Selenium; CAR, Carnosine.

A contributing mechanism behind the pathophysiology of sepsis involves mitochondrial dysfunction, which generates significant levels of ROS which can result in cell death (e.g., mitoptosis) (**Figure 3**) (Preau et al., 2021). By inducing mitochondrial decoupling, which in turn enhances harm from OS and sepsis, ROS and RNS exacerbate the damage in processes like sepsis. The pathogenesis of sepsis is attributed to an exaggerated immune and inflammatory reaction, marked by a substantial surge in ROS, nitric oxide (NO), and inflammatory cytokines. The overexpression of iNOS in sepsis may be inhibited by antioxidants, as nuclear factor-κB regulates iNOS expression and can be activated by ROS. Typically, intricate antioxidant defense mechanisms regulate OS in mitochondria through interdependent interactions. Sepsis results in a widespread impairment of the vascular endothelium, tissue function, and overall respiratory capacity. This is accompanied by a depletion of antioxidants and dysfunction in mitochondrial respiration, leading to reduced ATP and O_2_ consumption levels.

The pathogenesis of sepsis-associated organ dysfunction is linked to oxidative damage to mitochondria. Diminished ATP levels expedite apoptosis and necrosis and are a contributing factor to multiple organ failure. In particular, mitochondrial cardiolipin peroxidation increases ROS generation via cytochrome c dissociation, leading to decreased ATP levels (**Figure 3**) (Murphy, 2009). In addition, an oxidant environment can cause alterations in protein, lipid, and DNA structure, which in turn causes deficiency in ATP and further deterioration of homeostatic mechanisms (Rubio-Gayosso et al., 2006). Increased mitochondrial permeability is connected with mitochondrial dysfunction (Belikova et al., 2007). Sepsis leads to a reduction in the rates of mitochondrial state 3 respiration. Non-surviving septic patients exhibited lower ATP concentrations and ATP : ADP ratios compared to survivors (Brealey et al., 2002). Additionally, septic shock alters cytoplasmic glycolysis at the transcriptional level. For example, when healthy participants given intravenous endotoxin (Calvano et al., 2005), some of the most important metabolic enzymes involved in glycolysis and the mitochondrial respiratory chain (MRC) were found to be transiently under-expressed. Also, in the diaphragms of septic rats, transcription, synthesis, and activity of MRC components and the key enzyme of glycolysis, phosphofructokinase-1, were found to be downregulated (Callahan and Supinski, 2005). Moreover, in septic rat muscle, pyruvate dehydrogenase activity was reduced and its inhibitor, pyruvate dehydrogenase kinase activity, was increased, resulting in less pyruvate entering the mitochondria and more pyruvate being converted to lactate (Vary, 1996; Losser et al., 2010). Multiple investigations have shown that sepsis patients have cellular energy loss. In critically ill patients, this is linked to poorer outcomes, particularly mitochondrial dysfunction (Brown, 2001; Prauchner, 2017). This results in decreased levels of intracellular ATP leading to changes in Ca^2+^ homeostasis, further increase in ROS, and release of pro-apoptotic proteins. Concurrently, changes in mitochondrial permeability occur due to the correlation between matrix inflammation and respiratory chain uncoupling. The Ca^2+^ outflow, increased ROS production, membrane potential loss, and the release of cytochrome c from mitochondrial complexes occur within the mitochondria and contribute to cellular apoptosis (**Figure 3**) (Kozlov et al., 2011). Ultimately, apoptosis and cytochrome c release can be triggered by disruption to the mitochondrial membrane (**Figure 3**) (Galley, 2011). Another key player in the evolution of sepsis and OS is the family of NADPH oxidases (NOX). Seven different isoforms of NOX, each with its own unique catalytic domain, have been described. These include NOX1, NOX2, NOX3, NOX4, and NOX5 as well as Duox1 and Duox2. NOX4 is the most potent generator of ROS since it can be activated by various inflammatory stimuli such as LPS, tumor necrosis factor-alpha (TNF-α), TGF-β, and hypoxia (Pendyala et al., 2009).

Changes in cellular energy metabolism occur in sepsis due to disruptions in the mitochondrial electron transport and glycolytic pathways. Acute septic conditions are associated with changes in glucose metabolism that can be thought of as a “redistribution of glucose consumption away from mitochondrial oxidative phosphorylation” toward other metabolic pathways, including lactate generation. There appears to be no impact on cellular energy supply from this rerouting. This could be because the cells are consuming less ATP, which causes them to show signs of metabolic failure (Singer, 2005). The development and maintenance of sepsis-induced anomalies in cellular energy metabolism can be attributed to the decline in the expression of genes that encode crucial electron transport and glycolytic proteins (Callahan and Supinski, 2005). The mitochondrial respiratory chain (MRC) undergoes structural and functional changes during sepsis (Losser et al., 2010), with essential enzymes of electron transport and ATP production (Levy, 2007) and mitochondrial biogenesis (Haden et al., 2007) being inhibited. These findings were also observed in monocytes derived from patients in septic shock (Adrie et al., 2001) and skeletal muscle (Brealey et al., 2002). Sepsis results in the suppression of genes that encode components of the mitochondrial electron transport chain (ETC), including cytochrome-c oxidase 5A and the rate-limiting enzyme for glycolysis, phosphofructokinase (PFK). Peroxiredoxin-3 and thioredoxin reductase-2 enzymes, which are involved in the mitochondrial thioredoxin-2 (TRX-2) oxidation, are part of yet another antioxidant defense system. The TRX-2 system proteins exhibited greater resistance to OS and played a significant role in safeguarding against mitochondrial dysfunction under sepsis conditions in human endothelial cells *in vitro* studies (Galley, 2011), suggesting that the TRX-2 system plays a larger role in sepsis.

The involvement of mitochondrial OS in the development of organ dysfunction induced by sepsis has been established. In simulated sepsis conditions, the silent information regulator-1 (SIRT-1)-activator-3 (a selective synthetic agonist of SIRT-1) activation of the peroxisome proliferator-activated receptor gamma co-activator 1-alpha (PGC1α) and nuclear factor erythroid 2-like 2 (NFE2L2) pathways demonstrated a protective effect on cells against LPS/PepG-induced loss of mitochondrial membrane potential and metabolic activity, as well as a reduction in interleukin-6 (IL-6) responses. Furthermore, there was an observed increase in mitochondrial biogenesis and glutathione (McCreath et al., 2016).

## 4 Sepsis and inflammatory reactions

Both anti-inflammatory and pro-inflammatory cytokines are believed to have significant roles in sepsis pathogenesis. Septic individuals exhibited notable increases in plasma and/or serum concentrations of IL-6, interleukin-8 (IL-8), interleukin-10 (IL-10), interleukin-18 (IL-18), and TNF-α. The overproduction of IL-6, IL-8, IL-18, and TNF-α contributes to excessive inflammation, while IL-10 is involved in later immunosuppression (Chaudhry et al., 2013). Multiple studies have demonstrated a positive correlation between elevated plasma concentrations of IL-18 and unfavorable clinical prognosis in septic individuals (Tschoeke et al., 2006). Similarly, numerous reports note elevation in serum concentrations of IL-1β, IL-6, IL-8, and TNF-α between neonatal individuals with sepsis and the corresponding control cohorts. Subsequent antibiotic treatment resulted in a noteworthy reduction in the serum levels of these cytokines, underscoring the efficacy of antibiotic intervention in mitigating the inflammatory response associated with sepsis in neonates (Kurt et al., 2007). Conversely, the prediction of mortality at 28 days relied specifically on the utilization of IL-8 and MCP-1. The cytokines IL-8 and MCP-1 showed the most significant correlation with the sequential organ failure assessment (SOFA) scores in septic patients on the first day of observation.

**Figure 4:**
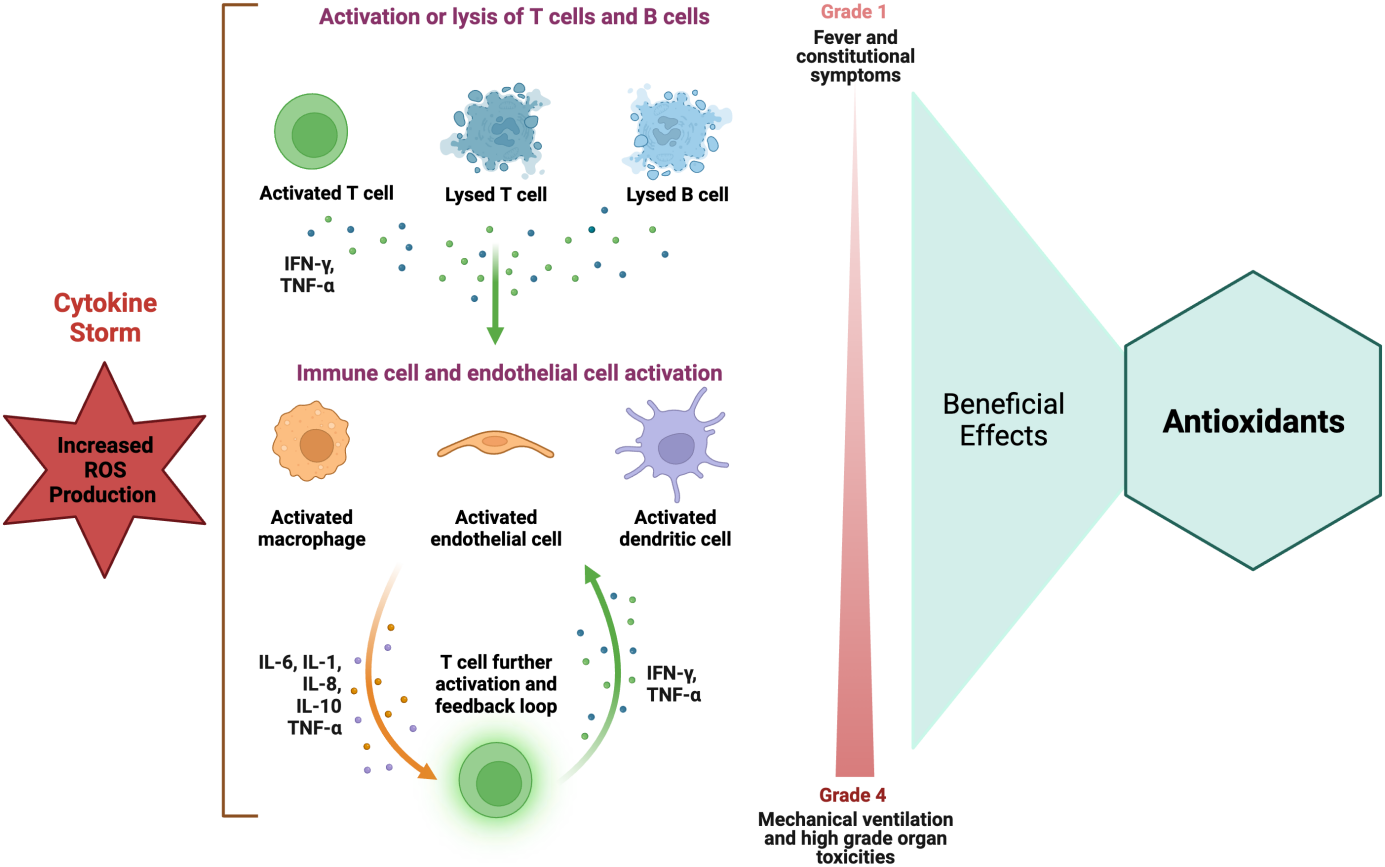
Role of antioxidants in hyper-inflammatory immune response in sepsis. Elevated ROS level is strongly associated with the occurrence of increased inflammatory responses, particularly those characterized by a cytokine storm. Antioxidants have been recommended as a potential therapy for suppressing the immune response in cases of increased inflammatory reactions. The figure was generated using BioRender (www.biorender.com; accessed on 1st Nov 2023). IFN-γ, Interferon-gamma; IL, interleukin; TNF-α, tumor necrosis factor-alpha.

Despite the development of new therapeutic agents, sepsis continues to exhibit high rates of morbidity and mortality; therefore, it is important to understand its pathogenesis in order to devise efficacious treatment modalities. For many years, it was believed that the pathophysiology of sepsis was associated with uncontrolled inflammation (i.e., cytokine storm) (**Figure 4**), resulting in cardiovascular failure, organ failure, and mortality. There has been a significant increase in the testing of various therapeutic agents, such as tumor necrosis factor inhibitors, IL-1 blockers, and corticosteroids, in large-scale clinical trials, with the aim of targeting inflammation. No cytokine-targeting agents or anti-inflammatory treatments have demonstrated substantial efficacy, and certain interventions have led to higher mortality rates. Researchers have discovered that the inflammatory response profiles in individuals with sepsis are more intricate than initially believed (Hotchkiss et al., 2013). Research findings have demonstrated that there exists a significant suppression in the production of cytokines by splenocytes obtained postmortem from individuals who succumbed to sepsis. This suppression can be attributed to a phenomenon known as T-cell exhaustion, which is a recently acknowledged mechanism of immunosuppression that arises as a result of prolonged exposure to antigens (Hotchkiss et al., 2013).

Numerous clinical trials have been conducted to investigate the efficacy of TNF and interleukin 1 (IL-1) antagonists, toll receptor blockers, and endotoxin antagonists in the context of sepsis. The outcomes of over 30 trials involving a range of anticytokine and anti-inflammatory medications have consistently demonstrated a lack of favorable effects or, in certain instances, a decrease in survival rates. The findings collectively suggest that certain individuals afflicted with sepsis exhibit a rapid synthesis of both proinflammatory and anti-inflammatory cytokines, while others demonstrate an imbalance in favor of anti-inflammatory cytokines or a general suppression of cytokine production. Indeed, the precise etiology underlying mortality and organ dysfunction in the majority of septic patients remains elusive. Clinical trials place significant emphasis on the extent of immunosuppression observed in cases of sepsis while also elucidating the reasons behind the lack of success in numerous sepsis trials that focused on inhibiting inflammatory mediators or disrupting pathogen recognition signaling pathways. Also, the occurrence of sepsis elicits robust inflammatory responses, which in turn trigger alterations in gene expression through epigenetic mechanisms encompassing DNA methylation, histone modification, and chromatin remodeling. These processes are collectively responsible for inducing both upregulation and downregulation of gene activity. The findings from various studies suggest that there is a correlation between intense immunoinflammatory responses, such as sepsis, and the occurrence of epigenetic changes. These changes have been observed to lead to a disruption in the expression of genes responsible for regulating crucial immune activation responses and, consequently, rendering the host more vulnerable to infections. The salient observation persists that a considerable number of ICU patients fail to recover due to the persistence of an ongoing infection. Despite the administration of broad-spectrum antibiotics and the implementation of rigorous source control measures, a number of patients fail to completely eliminate their infections and subsequently experience the onset of secondary hospital-acquired infections (Otto et al., 2011; Kethireddy and Kumar, 2012). Hence, it can be postulated that therapeutic interventions aimed at enhancing immune competence may exert a profound impact on clinical outcomes, manifesting in expedited resolution of the primary infection and averted morbidity and mortality resulting from secondary infections of a fatal nature.

A body of evidence has substantiated the notion that a deficiency in micronutrients can cause a state of immune function suppression. This phenomenon exerts its influence on both the innate T-cell-mediated immune response and the adaptive antibody response, thereby inducing modifications to the equilibrium of the host’s response. The maintenance of an optimal immune system function appears to necessitate the consumption of a sufficient quantity of vitamins and antioxidant compounds (Mishra et al., 2023). The phenomenon of immunological alterations during the process of aging as a result of inadequate intake of essential micronutrients is well-documented. These alterations have been observed to occur with notable frequency and have been found to be closely linked to an increased vulnerability to various infectious agents. OS triggers inflammatory responses through the activation of redox pathways due to nuclear factor κB (NFκB) activation, leading to elevated levels of circulating inflammatory mediators such as cytokines and pentraxin-3 in sepsis patients (Arnalich et al., 2000; Galley, 2011).

While ROS are integral to cellular processes, especially those of immune cells, it is imperative to maintain sufficient levels of antioxidant defenses to counteract the deleterious consequences of excessive ROS generation. In addition to their role in generating ROS essential for microbicidal activity, immune cells exhibit susceptibility to exogenous ROS as a consequence of their elevated polyunsaturated fatty acids (PUFA) composition. Immune cells exhibit an atypical nature in comparison to other somatic cells due to their elevated concentrations of antioxidant vitamins. This characteristic is believed to confer a safeguard against LPx and immunosuppression, which are widely recognized hazards associated with increased PUFA levels (Bendich, 1988). The maintenance of an efficient immune response is facilitated by the presence of antioxidant vitamins and trace elements (Wintergerst et al., 2007). This was evident from a study in which the administration of a vitamin E supplement to a cohort of elderly healthy individuals resulted in an elevation in the antibody titer for both the hepatitis B and tetanus vaccines. Consequently, the supplementation led to an augmentation of T-cell-mediated functions (Simin Nikbin et al., 1997). The maintenance of sufficient levels of antioxidants may present a valuable strategy in mitigating cellular damage and impairment observed in select inflammatory and autoimmune conditions (De la Fuente, 2002; De La Fuente et al., 2005).

## 5 Antioxidants with antimicrobial properties in sepsis treatment

### 5.1 Antimicrobial effects of vitamin E

Reports have demonstrated some efficacy of vitamin E against infectious diseases, specifically respiratory infections, chlamydiosis, and bacterial infections caused by *E. coli* and *Helicobacter pylori* (Hayek et al., 1997; Melis et al., 2009; Sezikli et al., 2009; Hartmann et al., 2020). Additionaly, tocotrienol derivative (shares properties of vitamin E)? extracted from *Tovomitopsis psychotriifolia* leaves was found to inhibit the growth of Gram-positive bacteria (*Bacillus cereus* and *S. aureus*) and Gram-negative bacteria (*P. aeruginosa*) (Setzer et al., 1995). The direct antimicrobial effects of a water-soluble vitamin E derivative (α-tocopheryl-polyethylene-glycol-succinate; TPGS), commonly used in drug delivery systems such as liposomes, micelles, prodrugs, and nanoparticles due to its amphiphilic structure (Kang et al., 2019) are attributed to its ability to disrupt bacterial cell membranes, allowing antimicrobial agents to penetrate more easily (Andrade et al., 2014).

### 5.2 Antimicrobial properties of vitamin C

The potential antibacterial properties of vitamin C may vary depending on the concentration and the bacterial strain. Vitamin C has demonstrated significant antimicrobial efficacy against various pathogens, including *Campylobacter jejuni* (Mousavi et al., 2020), *Helicobacter pylori* (Zojaji et al., 2009), *Mycobacterium tuberculosis* (Hartmann et al., 2020), *E. faecalis*, *S. aureus*, *P. aeruginosa*, *Salmonella* and effectively counteract biofilm formation by methicillin-resistant *S. aureus* (MRSA) (Mousavi et al., 2019; Hartmann et al., 2020). Co-administration of vitamin C and quercetin had synergistic antibacterial effects (Kallio et al., 2012), whereas vitamin C with natural extracts like pomegranate rind extracts and white tea increases *S. aureus* suppression (Holloway et al., 2011). The experimental administration of tuberculosis sputum to guinea pigs with a deficiency in vitamin C resulted in the manifestation of intestinal tuberculosis; in comparison, guinea pigs provided tomato juice containing vitamin C did not exhibit any signs of the disease (McCONKEY and Smith, 1933). Vitamin C, combined with lactic acid, inhibits the replication of the *E. coli* O157:H7 strain while in combination with deferoxamine, inhibits Gram-positive cocci (e.g., *S. epidermidis* and *S. aureus*) and Gram-negative bacilli (e.g., *E. coli*, *K. pneumoniae*, and *Proteus mirabilis)* (van Asbeck et al., 1983). The synergistic potential of vitamin C in augmenting the antibacterial properties of various agents, including epigallocatechin gallate, has been observed, even extending its efficacy to combat multidrug-resistant MRSA (Hatano et al., 2008). The inhibitory impact of vitamin C on the *in vitro* growth of *C. jejuni* is primarily attributed to the presence of vitamin C oxidation byproducts, namely L-dehydroascorbic acid or L-diketogulonic acid (Mousavi et al., 2019).

Vitamin C, particularly in the dehydroascorbic acid (DHA) form, exerts inhibitory effects on the replication of poliovirus type 1, influenza virus type A, and herpes simplex virus type 1 (Kim et al., 2016). The observed decrease in parasite burdens in mice infected with *Trypanosoma cruzi* and *Plasmodium yoelii* 17XL following the administration of vitamin C could potentially be attributed to the immunomodulatory characteristics inherent to vitamin C (Puente et al., 2018; Mousavi et al., 2019). An inhibition of Hsp90-mediated morphogenesis in *Candida albicans* due to the association with Vitamin C has also been reported. At the very least, in part, the powerful antibacterial effects of vitamin C can be attributed to its low pH and, as a result, its milieu-modifying characteristics (Mousavi et al., 2019).

### 5.3 Antimicrobial properties of melatonin

Melatonin (N-acetyl-5-methoxytryptamine), an indoleamine, has a strong ability to bind to metals, including iron responsible for its antibacterial properties (He et al., 2021). The molecular mechanisms underlying the antimicrobial actions of melatonin have been postulated to arise from its impact on various cellular processes. These include the modulation of free radical generation, direct regulation of bacterial replication, and depletion of intracellular substrates such as iron, among others (Srinivasan et al., 2010, 2012). Melatonin can effectively traverse biological barriers, including the bacterial cell wall, and inhibit bacterial growth by binding to free iron in the cytoplasm. Of note, iron is very important for bacteria, rendering the emergence of bacterial resistance considerably challenging for those lacking access to iron. The inner membrane of gram-positive bacteria and the inner and outer membranes of gram-negative bacteria contain high levels of phospholipids. The inhibitory effects of melatonin on the uptake of total fatty acids have been demonstrated, suggesting its potential efficacy in impeding the proliferation of rapidly dividing prokaryotic organisms (Tekbas et al., 2008). The administration of melatonin yielded a substantial reduction in the lipid content of *Saccharomyces cerevisiae*. The efficacy of melatonin as a potent agent in the reduction of lipid levels in *Candida albicans* also has been demonstrated (Konar et al., 2000).

### 5.4 N-acetylcysteine (NAC) antimicrobial properties

The efficacy of N-acetylcysteine (NAC) in impeding biofilm formation and proliferation by non-oral pathogens, including *Stenotrophomonas maltophilia*, *Staphylococcus* species, *P. aeruginosa*, and *Burkholderia cepacia* as well as by oral pathogens, including *Porphyromonas gingivalis*, *Streptococcus mutans*, *Aggregatibacter actinomycetemcomitans, P. intermedia*, and *E. faecalis* (Abdulrab et al., 2022). The antibiofilm and antibacterial effects of NAC are likely attributed to its ability to diminish biofilm formation, impede bacterial adherence, and diminish the synthesis of extracellular polysaccharide matrix. The therapeutic efficacy of NAC is attributed to its thiol group, which serves as the active moiety responsible for scavenging free radicals and disrupting disulfide bonds within bacterial proteins. This process ultimately culminates in the irreparable impairment of bacterial growth (Olofsson et al., 2003).

### 5.5 Selenium antimicrobial properties

The antimicrobial activity of selenium nanoparticles (SeNPs) has been reported against *E. coli*, *S. aureus*, and *Mycobacterium tuberculosis* (*M. tuberculosis*) (Guisbiers et al., 2016; Martínez-Esquivias et al., 2021). SeNPs exhibit a propensity for adhering to the cellular membrane and subsequently permeating the bacterial cell, thereby instigating detrimental effects on the structural integrity of the membrane. This deleterious outcome arises from the generation of ROS, which in turn triggers the demise of the bacterial entity. SeNPs also exhibit notable antifungal activity against *Aspergillus* species, *Fusarium anthophilum* and *Candida albicans*, with a higher degree of efficacy in inhibiting fungal growth compared to their antibacterial properties (Lara et al., 2018; Gunti et al., 2019; Martínez-Esquivias et al., 2021).

### 5.6 Antimicrobial properties of carnosine

The antiviral efficacy of dipeptide carnosine primarily occurs through the suppression of viral genome replication and the prevention of viral entry into host cells as studied against Dengue virus (DENV) serotype 2 and Zika virus (ZIKV) (Rothan et al., 2019). It has been observed that both DENV and ZIKV utilize common receptors, specifically HSP90/HSP70, in order to facilitate their entry into host cells (Cruz-Oliveira et al., 2015; Sirohi and Kuhn, 2017). The plausibility arises from the potential of carnosine to engage in competitive binding with HSP70, thereby exerting inhibitory effects that could result in diminished viral infection. It has been observed that polaprezinc (a zinc-chelated L-carnosine compound) exhibits the ability to bind to and effectively inhibit the activity of heat shock protein 70 (HSP70) (Haga et al., 2013). The computational analysis yielded predictions regarding the potential interaction between carnosine and the viral NS2B-NS3 serine protease, as well as the viral envelope. These interactions have the potential to impede viral genome replication and hinder viral entry.

The utilization of carnosine within a vitamin K3 carnosine peptide (VKC) was explored (Kandhasamy et al., 2021), with VKC displaying considerable effectiveness in combating microbial activity, particularly against Gram-negative (*P. aeruginosa* and *E. coli*), and Gram-positive (*S. aureus*) bacterial strains. However, the observed antimicrobial properties of VKC were reported to be attributed to the presence of quinone moieties, which induce perturbation and disruption of functional mechanisms in the bacterial cell, thereby facilitating comprehensive inhibition of microbial growth. In the contrary, according to Gholibegloo et al. (2018), it has been reported that carnosine possesses the capability to enhance the antibacterial efficacy of graphene oxide in its action against *Streptococcus mutans* (Gholibegloo et al., 2018).

### 5.7 Antimicrobial properties of fish oil

The antibacterial activity of polyunsaturated fatty acids found in fish oil has been observed to exhibit efficacy against a range of foodborne pathogens, including *Salmonella* and pathogenic *E. coli* (Dhakal and Aldrich, 2022). Fatty acids have been documented to induce modifications in the hydrophobicity of cell membranes, the charge of cell surfaces, and the integrity of membranes. These alterations subsequently give rise to electron leakage, ultimately culminating in cell death (Desbois and Smith, 2010; Chanda et al., 2018). Docosahexaenoic acid (DHA) and eicosapentaenoic acid (EPA) are recognized as the primary constituents of the omega-3 polyunsaturated fatty acids (ω-3 PUFAs) that exhibit notable antimicrobial properties (Chanda et al., 2018). The occurrence of membrane disruption and the subsequent likelihood of cell lysis in bacterial cells following exposure to EPA has been documented in previous studies. *In vitro* studies have demonstrated the antimicrobial and anti-biofilm properties of EPA and DHA against various strains, including *S. epidermidis*, *S. aureus*, and *P. aeruginosa* and also against multi-drug resistant strains of *S. aureus* and coagulase-negative Staphylococci, obtained from periprosthetic joint infection patients (Coraça-Huber et al., 2021).

## 6 Antioxidant treatment

The impact of various therapeutic agents on the management of sepsis has been investigated. Antioxidant therapy is widely used in cases of OS damage. Antioxidants come in several forms, each with its own unique mechanism of action and clinical use (Sahoo et al., 2008b; Prauchner, 2017; Chainy and Sahoo, 2020; Toro-Pérez and Rodrigo, 2021; Ilango et al., 2022; Patani et al., 2023; Sahoo and Chainy, 2023; Sahoo et al., 2023a). Although oxidative damage is commonly observed in various organs during the progression of sepsis and there exists a direct association between oxidative markers and organ damage, the efficacy of antioxidant effects appears to be contingent not only on the reduction of oxidative damage but also on its anti-inflammatory properties.

The administration of antioxidants has effectively reversed organ failure in some murine sepsis models. This phenomenon can be attributed to the imbalance of antioxidant enzymes, infiltration of neutrophils, and the presence of OS. In one murine study, the group with cecal ligation and puncture (CLP) exhibited a positive correlation between the levels of TBARS and markers of organ injury in the lung and kidney. While the correlation between oxidative damage and an elevated SOD/CAT ratio was observed solely in the pulmonary system (Andrades et al., 2011), the correlation between MPO activity and oxidative damage was observed in the kidney but not in the lung, indicating that the origin of oxidative damage varies depending on the organ. These findings demonstrate variations in the impact of fundamental support and antioxidants on organ dysfunction subsequent to sepsis (Andrades et al., 2011).

Antioxidant nutrients, such as vitamins C and E, β-carotene, selenium, iron, copper, and zinc, are frequently incorporated into dietary regimens (**Table 2**). These nutrients enhance diverse immune functions, thereby playing a significant protective role against infections instigated by bacteria, viruses, or parasites. Consequently, it is plausible that interventions aimed at bolstering host immunity could potentially augment survival rates (Puertollano et al., 2011). Moreover, during OS occurring in sepsis, three primary mechanisms play a pivotal role: vasomotor impairment, mitochondrial dysfunction, and necrosis in various tissues (Abelli et al., 2022). Hence, an overabundance of ROS gives rise to a disturbance in the physiological endothelial function, resulting in vasoplegia, an excessive procoagulant state induced by the inhibition of the ADAMTS-13 (a disintegrin and metalloproteinase with thrombospondin motifs 13) enzyme, and impairment of mitochondrial integrity, thereby disturbing the signaling cascades governing thrombosis and inflammation (Dolmatova et al., 2021). There exist certain antioxidant molecules that possess the capability to selectively target and modulate the aforementioned reported mechanisms. Consistent with this notion, a variety of antioxidants, including polyphenols, β-glucan, vitamins C and E, melatonin, N-acetylcysteine, mitochondrion-targeted antioxidants (MitoQ, MitoVitE, and peptides linked to dimethyl tyrosine), selenium salts, and organoselenium compounds, have demonstrated efficacy in mitigating OS in animal models of sepsis, as well as in several clinical trials involving septic patients. However, it is essential to note that there have been studies on antioxidant therapies in which adverse effects have been observed (Jain and Chandel, 2013).

**Table 2:** Antioxidants reduce oxidative stress (OS) as a potential sepsis treatment.

Antioxidant	Antioxidant Effects (ROS/RNS Scavenging activity)	Potential Benefits in Sepsis	Models	Reference
N-Acetylcysteine (NAC)	MDA↓	• Reduces cell damage • Maintains protein structure and function • Reduces tissue injury and inflammation • Aids in the removal of hazardous substances • Improves antioxidant defense	• *In vitro* studies using A549 cells murine models • NAC in TB treatment in humans from 1960 to 31 May 2022	(Pei et al., 2018; Zukowski et al., 2018; Shiozawa et al., 2020; Tenório et al., 2021; Mapamba et al., 2022)
	PC↓			
	MPO↓			
	GST↑			
	GSH↑			
Vitamin C (Ascorbic Acid)	ROS↓	• Acts as an effective ROS scavenger • Reduces LPx • Stops protein oxidation • Reduces the OS caused by neutrophils • Promotes GST activity and detoxification • Cofactor for antioxidant enzymes	• Murine CLP model • Murine renal and intestinal ischemia- reperfusion model	(Tyml et al., 2005; Azari et al., 2015; Kumar et al., 2022; Chaudhary et al., 2023)
	RNS↓			
	MDA↓			
	PC↓			
	MPO↓			
	GST↑			
	GSH↑			
Vitamin E (Tocopherol)	ROS↓	• As a free radical scavenger, it neutralizes ROS/RNS • Prevents LPx and MDA production • Protein oxidation and carbonylation are reduced • Restricts the effects of MPO’s pro-oxidative activities • Increases intracellular GSH levels, assisting in the removal of ROS	• Acute ovine model • Murine CLP model	(Atli et al., 2012; Li et al., 2015; Bergin et al., 2021; Thompson et al., 2022)
	RNS↓			
	MDA↓			
	PC↓			
	MPO↓			
	GST↑			
	GSH↑			
Selenium	MDA↓	• Boosting the immune system • Anti-inflammatory actions • Improved wound healing • Better microcirculation • Organ protection potential	• PCV2 mouse model	(Liu et al., 2015; Sygitowicz and Sitkiewicz, 2020; Mahmoodpoor et al., 2022)
	PC↓			
	MPO↓			
	GSH↑			
Zinc	NO↓	• Aids in antioxidant defense and detoxification within the cell • Promotes the breakdown of superoxide radicals • Antioxidant capabilities reduce LPx	• Porcine model of sepsis • Porcine jejunal epithelial cell line IPEC-J2 *in* * vitro* studies	(Hoeger et al., 2015; Marreiro et al., 2017; Rajoka et al., 2021; Zhang M. et al., 2021)
	SOD↑			
	CAT↑			
	GPx↑			
	GR↑			
	MDA↓			
	PC↓			
Melatonin	MDA↓	• Free radical scavenging and OS reduction • Modulates inflammatory responses, which are involved in sepsis • Protects the mitochondria from dysfunction, which can occur in sepsis • Controls immunological responses and prevent excessive inflammation • Aid in the function of blood vessel lining cells, which is crucial in sepsis	• Murine model of LPS-induced sepsis • Human umbilical vein endothelial cell (HUVEC-C) sepsis model	(Lowes et al., 2011; Tóthová and Celec, 2017; Biancatelli et al., 2020; Zhang et al., 2020)
	PC↓			
	MPO↓			
	GSH↑			
	NO↓			
	SOD↑			
	CAT↑			
	GPx↑			
	GR↑			
Alpha-Lipoic Acid	MDA↓	• Neutralizes the detrimental reactive oxygen species (ROS) and free radicals produced by sepsis • Inhibits cytokine production and inflammatory pathways • Supports the health and energy generation of the mitochondria, possibly lowering cellular stress • Endothelial cell protection and vascular integrity • Increases cellular glutathione levels, a crucial antioxidant molecule	• LPS-induced endotoxemic model in rats	(Sung et al., 2005; Petronilho et al., 2016; Jomova et al., 2023)
	PC↓			
	MPO↓			
	GSH↑			
	Nrf2↑			
	HO-1↑			
Quercetin	MDA↓	• Decreases pro-inflammatory cytokines (such as TNF-α, IL-6) production and inhibiting the overall inflammatory response, which is a significant factor in sepsis progression • Enhanced vasodilation and blood pressure • Immune modulation • Vascular integrity improvement in sepsis • Reduce inflammation and OS in the gut lining to maintain gut barrier integrity, potentially decreasing bacterial translocation and endotoxemia in sepsis	• Murine model of exercise-induced susceptibility to influenza H1N1	(Marik, 2020; Sun et al., 2021; Enayati et al., 2022)
	PC↓			
	MPO↓			
	GST↑			
Glutathione	SOD↑	• Plays a pivotal role in the detoxification of deleterious substances, the removal of metabolic waste, and the potential reduction of organ damage in sepsis • Anti-inflammatory properties • Its cell-protective properties and capacity to lower inflammation may aid in tissue regeneration and help speedier recovery from sepsis • Glutathione collaborates with other antioxidants, such as vitamins C and E, to provide a comprehensive defense against oxidative damage in sepsis	• Murine embryos model • Lipopolysaccharide- peptidoglycan rat model	(Lowes et al., 2008; Förstermann et al., 2017; Memarzia et al., 2021; Rahman et al., 2022)
	CAT↑			
	GPx↑			
	GR↑			
	MDA↓			
	PC↓			
Carnosine	PC↓	• Carnosine has antioxidant capabilities and can scavenge reactive oxygen and nitrogen species • It also forms complexes with metal ions (iron, cobalt, copper and zinc) protecting cells against damage • Immune system modulation helps balance the immune response in sepsis, preventing both an overactive and an inadequate immune reaction	• Rat CLP model of sepsis • *In vitro* studies using murine RAW 264.7 macrophages	(Sahin and Donmez, 2018; Caruso et al., 2019; Kulczyński et al., 2019; Jukić et al., 2021)
	ROS↓			
	AGE↓			
Fish oil (rich in omega-3 fatty acids)	CAT↑	Omega-3 fatty acids function as an anti-inflammatory and potent antioxidant, has demonstrated a potential protective effect against organic dysfunction (liver injuries) and alterations caused by sepsis, particularly in oxidative stress conditions.	• Septic Wistar rats	(Velasque et al., 2023)
	GPx↑			
	Thiols↑			
	DCF↓			
	TBARS↓			

SOD, Superoxide dismutase; CAT, catalase; GPx, glutathione peroxidases; GR, glutathione reductase; GST, glutathione S-transferases; GSH, reduced glutathione; PC, protein carbonyl; MDA, malondialdehyde; MPO, myeloperoxidase; NO, nitric oxide; HO-1, heme oxygenase-1; Nrf2, nuclear factor erythroid 2–related factor 2; AGE, Advanced glycation product; CLP, Cecal ligation and perforation; PCV2, Porcine Circovirus Type 2; LPS, lipopolysaccharide; LPx, lipid peroxidation; DCF, 2’,7’-dichlorofluorescein; TBARS, thiobarbituric acid reactive substances. ↑, increased; ↓, decreased.

Some reports suggest that antioxidants can potentially interfere with the regular signaling mechanisms that regulate the response to severe infection. Increasing evidence suggests that NOX and mitochondrial-generated ROS play a crucial role in the optimal activation of lymphocytes and monocytes, which are essential for an effective response to infectious agents (Pearce and Pearce, 2013). Mitochondrial ROS production is important for the activation of multiple Toll-like receptors (TLRs) and the RIG-I-like receptors (RLRs) (West et al., 2011) as well as in the activation of inflammasomes (Tschopp and Schroder, 2010) and the activation and proliferation of T cells (Sena et al., 2013). The inflammasome induces proteolytic processing of proinflammatory cytokines by recognizing various PAMPs) and DAMPs. Hence, it is reasonable to suggest that the perturbation of this intricate mechanism of ROS by exogenous agents possessing antioxidant properties may not yield a noticeable amelioration in the overall prognosis of the organ implicated in the context of multiple organ failure. The physiological responses of inflammatory cells are influenced by various levels of ROS. Elevated intracellular ROS concentrations are closely linked to the manifestation of increased inflammatory responses, specifically those characterized by cytokine storm (**Figure 4**). Conversely, diminished ROS levels have been correlated with a hypoactive inflammatory response, which can subsequently result in immunosuppression. The presence of ROS within the intermediate range has been observed to exhibit a correlation with immune cell function within the normal range (**Figure 2**). Hence, the effectiveness of antioxidants is contingent upon the generation of ROS within inflammatory cells. The utilization of antioxidants has been postulated to yield advantageous outcomes in instances characterized by increased inflammatory reactions (**Figure 2**), yet it may prove to be deleterious in circumstances marked by a relative state of immunosuppression. Hence, the effectiveness of antioxidants is contingent upon an individual’s inflammatory response profile, wherein the precise timing and duration of antioxidant administration play a crucial role in manifesting a beneficial outcome (Jain and Chandel, 2013).

### 6.1 Melatonin

**Figure 5:**
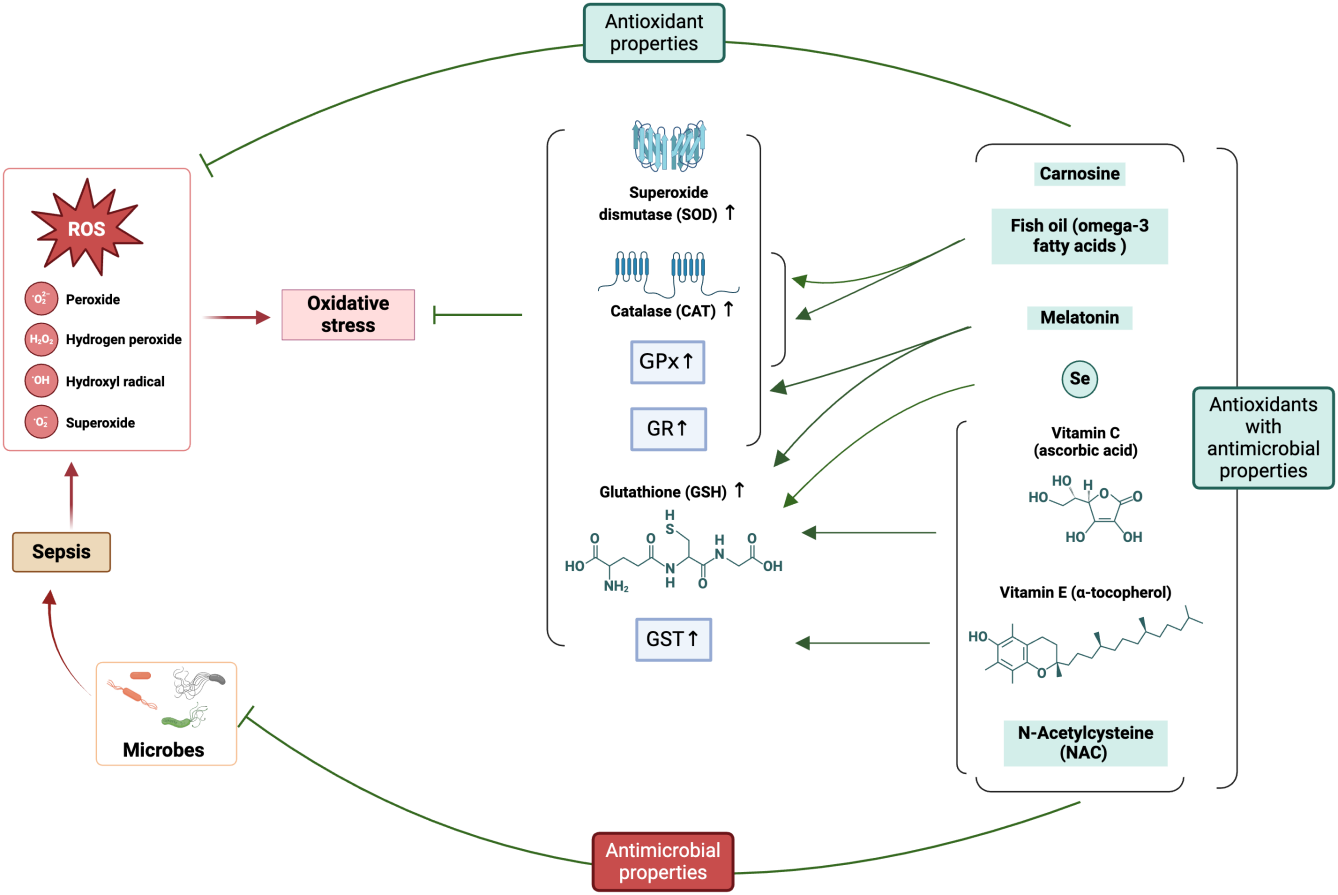
Involvement of ROS in sepsis pathophysiology and the potential application of antioxidants (with antimicrobial properties) ameliorating oxidative stress. The figure was generated using BioRender (www.biorender.com; accessed on 6th Feb 2024). SOD, Superoxide dismutase; CAT, catalase; GPx, glutathione peroxidases; GR, glutathione reductase; GST, glutathione S-transferases; GSH, reduced glutathione; Se, Selenium.

Melatonin has strong anti-inflammatory and antiapoptotic effects in addition to its role as an antioxidant scavenger for ROS and RNS (Srinivasan et al., 2010, Srinivasan et al., 2012). The potential protective effects of melatonin against sepsis (**Table 2** and **Figure 5**) are postulated to arise from its antioxidative, immunomodulatory, and inhibitory properties targeting the production and activation of pro-inflammatory mediators (Srinivasan et al., 2012). The anti-inflammatory properties of melatonin have been ascribed to its ability to inhibit nitric oxide synthase, resulting in a decrease in peroxynitrite production. Additionally, melatonin has been shown to stimulate various antioxidant enzymes, thereby bolstering the antioxidant defense system. Furthermore, melatonin exerts protective effects on mitochondrial function and serves as a preventive measure against apoptosis (Srinivasan et al., 2010). The injection of melatonin into LPS-treated rats lowered mitochondrial NOS activity and increased the activities of mitochondrial ETC complexes I and IV (Escames et al., 2003). In addition, melatonin protects mitochondria from damage caused by mitochondrial iNOS in septic mice (Escames et al., 2006) and ATP synthesis in the mitochondria can be revived (López et al., 2006). Several inflammatory and OS markers were decreased in healthy individuals after they were given melatonin prior to the infusion of LPS (Alamili et al., 2014). The treatment of septic infants with melatonin led to decreased levels of LPx products and has several positive effects (Gitto et al., 2001). Several investigations in animals have shown that melatonin has protective antioxidant and anti-inflammatory effects against LPS or cecal ligation and puncture (CLP)-induced septic shock (Sewerynek et al., 1995; Carrillo-Vico et al., 2005; Wu et al., 2008; Mantzarlis et al., 2017). The administration of melatonin and vitamin C demonstrated a notable amelioration in organ dysfunction, as evaluated by sequential organ failure assessment (SOFA) score, among individuals afflicted with septic shock. The observed outcome may potentially be linked to a reduction in the ratio of NO_3_^−^ to NO_2_^−^ and the levels of LPx (Aisa-Alvarez et al., 2020). This finding implies that a synergistic approach involving multiple antioxidant interventions is necessary to optimize the overall clinical prognosis.

### 6.2 Vitamins

Vitamins, as indispensable micronutrients, play pivotal functions in numerous biological pathways that are intricately associated with the occurrence and progression of sepsis. Included among these relevant biological mechanisms are anti-inflammatory and antioxidant actions, gene transcription regulation, protein and hormone biosynthesis, and energy production. Exogenous antioxidants, such as vitamins C and E (**Table 2** and **Figure 5**), have been cataloged as a cure for the permanent harm that OS can inflict, making them an appealing alternative.

#### 6.2.1 Vitamin C

Ascorbic acid (vitamin C) is a water-soluble antioxidant that plays a role in a wide variety of enzymatic and non-enzymatic reactions and acts as a cofactor for numerous biological reactions (Marik, 2020). Active tocopherol can be produced when ascorbic acid reacts with the oxidized tocopheroxyl radical bound to the membrane (Pisoschi and Pop, 2015). The pivotal role of vitamin C in the host’s immune response against infections is underscored by its multifaceted effects. These effects encompass the augmentation of bacterial eradication, safeguarding the host from oxidative damage, maintaining optimal mitochondrial and metabolic functionality, regulating the inflammatory cascade, mitigating organ impairment, and ameliorating the protracted immunosuppressive stage of sepsis (Moskowitz et al., 2018).

In rats subjected to polymicrobial sepsis by CLP, the increased expression of iNOS, cyclooxygenase-2 (COX-2), and TNF-α mRNAs and decreased reduced glutathione were suppressed in the liver by vitamin C treatment intravenously immediately after the CLP procedure (Kim and Lee, 2004). In septic rats, the administration of high-dose ascorbic acid through total parenteral nutrition (TPN) supplementation exhibits the potential to safeguard cells against free radical-induced damage and enhance overall survival rates (Long et al., 2003). Critically ill patients, particularly those with sepsis, organ failure, or who have undergone major surgery, exhibit significantly reduced levels of ascorbic acid, and this decrease is attributed to the presence of increased ROS (Schorah et al., 1996; Long et al., 2003). Plasma levels of ascorbic acid are significantly reduced after trauma and during infection, and even with the administration of 300 or 1000 mg/day of supplemented TPN, these levels do not return to normal. Long et al. (2003) recommended administering a daily dose of 3000 mg of ascorbic acid for a minimum of three days after experiencing severe stress from trauma and infection, as there is an elevated turnover and breakdown of ascorbic acid in such conditions (Long et al., 2003). In a research study, individuals receiving vitamin E and ascorbate exhibited notable reductions in the occurrence of pulmonary morbidity, a lower incidence of multiple organ failure, a decrease in 28-day mortality rates, a shorter duration of mechanical ventilation, and a decreased length of ICU stay when compared to those patients who received standard care (Nathens et al., 2002). In a randomized study involving 37 patients with burns covering over 30% of their total body surface area, it was found that administering high-dose ascorbic acid as an adjuvant within the first 24 hours after thermal injury significantly reduced the need for resuscitation fluids, improved body weight gain, wound edema, and severity of respiratory dysfunction, and decreased serum malondialdehyde (MDA) levels (Tanaka et al., 2000). The therapeutic effects of vitamin C, such as immunological modulation, microcirculatory support, and neuroprotection, are contingent upon the dosage administered (Huang et al., 2001). Parenteral administration of vitamin C significantly increases plasma and cellular levels of the vitamin by more than 70 times compared to oral dosing and has the potential to protect against or reverse various pathological changes that occur during sepsis (Padayatty et al., 2004). Another study on a group of trauma patients who received treatment with N-acetylcysteine, selenium, and vitamins C and E for a duration of 7 days also exhibited a lower incidence of infectious complications and organ failures (Porter et al., 1999).

It is worth mentioning that certain organisms, namely humans (specifically anthropoid primates), guinea pigs, and selected fish species, exhibit an inherent inability to synthesize vitamin C and can have a compromised stress response. This deficiency is attributed to a defective mutation present within the L-gulono-γ-lactone oxidase (GULO) gene, which encodes the pivotal enzyme responsible for facilitating the catalysis of the last rate-limiting step in the biosynthesis of vitamin C (Marik, 2020) thus blocking normal mammalian conversion of blood sugar into ascorbate in the liver. It stands to reason that vitamin C (also referred as a stress hormone) supplementation would be helpful in sepsis, given that “Homo Sapiens Ascorbicus” cannot synthesize vitamin C and that dietary vitamin C levels are rapidly depleted in sepsis. The depletion of vitamin C in critically ill patients with sepsis is associated with increased organ dysfunction and mortality and is known to be dose-dependent (9). The correlation between outcomes and this association can be elucidated by its pleiotropic effects on biological pathways relevant to sepsis. Restoring the stress response and increasing survival in stressed humans could be achieved with treatment with vitamin C.

Further evidence that vitamin C is involved in the stress response (Padayatty et al., 2007; Marik, 2020) comes from the observation of very high levels of vitamin C in the adrenal gland and its release in response to adrenocorticotrophic hormone (ACTH). The stress response in mammals is characterized by increased vitamin C synthesis and secretion as well as activation of the hypothalamic-pituitary-adrenal axis (HPA-axis) and sympathoadrenal system (SAS). By comparing wild-type and GULO-/- knockout mice in a CLP sepsis model, it was noted that the former group performed better (Gao et al., 2017). However, when both groups of mice were given parenteral vitamin C, mortality rates dropped significantly. This research shows that sepsis patients could benefit from both naturally occurring vitamin C and therapeutic doses of vitamin C. In guinea pigs with low vitamin C levels, it was shown (Marik, 2020) that endotoxin was fatal.

Research shows that all patients with sepsis had low levels of vitamin C, with 40% having levels consistent with “latent scurvy.” (Carr et al., 2017; Marik and Hooper, 2018). These results show that vitamin C has a wide variety of synergistic effects when taken with other medicines that have favorable biological effects on sepsis. The effects encompass its role as an enzymatic cofactor in the synthesis of cortisol, catecholamines, and vasopressin. Additionally, it serves to scavenge ROS, maintain adequate capillary blood flow, and preserve arteriolar responsiveness to vasoactive medications by modulating redox-sensitive pathways. Furthermore, it safeguards against endothelial dysfunction and augments lung epithelial barrier function through nitric oxide-dependent mechanisms. It also plays a crucial role in regulating alveolar fluid clearance by inducing the expression of various protein channels, including cystic fibrosis transmembrane conductance regulator, aquaporin 5, epithelial sodium channel, and Na-K-ATPase. Lastly, it enhances the functionality of neutrophils and lymphocytes while concurrently downregulating pro-inflammatory pathways (Carr and Maggini, 2017).

The efficacy of orally administered vitamin C in the treatment of sepsis and critical illness has been a subject of research for several decades. The appropriate daily intake of vitamin C for individuals varies based on factors such as age, gender, and pregnancy status. Studies have demonstrated that the administration of vitamin C through parenteral means results in a significant increase in both plasma and cellular levels of the vitamin, exceeding those achieved through oral dosing by more than 70 times. This method of administration may offer protection against or aid in the restoration of various pathological changes that arise during sepsis (Padayatty et al., 2004).

Furthermore, it is worth noting that vitamin C has been associated with a range of systemic side effects, including but not limited to nausea, vomiting, fatigue, irritability, and coagulation abnormalities (Abelli et al., 2022). In relation to the renal system, there appears to be an association between vitamin C and elevated risks of acute kidney injury (AKI) and mortality during hospitalization (McCune et al., 2021). Conversely, the majority of research endeavors employ supraphysiological dosages of vitamin C with the intention of mitigating complications associated with sepsis. Elevated dosages of vitamin C have been correlated with an augmented transformation of the compound into calcium oxalate, exhibiting a dose-dependent pattern, thereby potentially resulting in the development of calcium oxalate nephropathy (Abelli et al., 2022). A randomized, placebo-controlled trial involving 872 patients conducted to investigate the effects of intravenous vitamin C on adults with sepsis receiving vasopressor therapy in the ICU showed that individuals who received intravenous vitamin C had a greater likelihood of experiencing death or persistent organ dysfunction at 28 days compared to those who received a placebo (Lamontagne et al., 2022).

However, hydrocortisone, ascorbic acid, and thiamine (HAT therapy), appear to have synergistic positive effects (Marik, 2018; Moskowitz et al., 2018). When administered as directed, HAT therapy substantially lowers the danger of acute renal (Marik, 2018). Patients with sepsis and septic shock may benefit from treatment with HAT therapy, which may lessen the likelihood of organ failure and death (Marik et al., 2017), as early evidence suggests that HAT therapy may lessen the risk of death or serious injury in individuals with sepsis and septic shock. Multiple randomized clinical trials (RCTs) have been conducted to examine the efficacy of HAT therapy in diverse cohorts of patients with sepsis (Marik, 2018; Na et al., 2021). HAT therapy has been observed to yield a notable enhancement in the SOFA score within the initial 72-hour timeframe while concurrently leading to a reduction in the duration of vasopressor administration among individuals suffering from sepsis. However, based on the negligible disparity in the average alteration of the SOFA score, it is evident that no discernible advantage in terms of mortality has been ascertained (Na et al., 2021).

#### 6.2.2 Vitamin E

Natural vitamin E, an efficacious lipid-soluble antioxidant, comprises a composite of eight chemically disparate entities, specifically α-, β-, γ- and δ-tocopherols, as well as α-, β-, γ- and δ-tocotrienols (Sen et al., 2007). Vitamin E, specifically α-tocopherol, being an endogenous antioxidant, functions by interrupting the chain reactions initiated by free oxygen radicals through the process of hydrogen atom donation, thus effectively safeguarding cells from the detrimental effects of ROS (Sahoo et al., 2008b, 2019; Chainy and Sahoo, 2020; Sahoo and Chainy, 2023). While the incorporation of free α-tocopherol and α-tocopherol acetate into alveolar macrophage membranes is limited *in vitro*, resulting in negligible impact on cellular activation, α-tocopherol succinate exhibits enhanced solubility and rapid cellular uptake *in vitro* (Bulger and Maier, 2003). The effectiveness of α-tocopherol succinate is attributed to its localization within the cell membrane, which allows for efficient antioxidant activity as the succinate component of the molecule is cleaved by cellular esterase activity, thereby revealing the active antioxidant site (Bulger and Maier, 2003). The α-tocopherol succinate pretreatment effectively inhibits the procoagulant activity of TNF induced by LPS and the production of prostaglandin E2 by macrophages derived from tissues (Mendez et al., 1995). Inhibition of the transcription of the TNF gene and the upregulation of NF-κB in the nucleus was observed by α-tocopherol succinate treatment. Furthermore, α-tocopherol succinate effectively inhibits the nonenzymatic peroxidation of membrane lipids induced by LPS, as indicated by the reduction in F2-isoprostane production (Bulger and Maier, 2003).

Vitamin E is essential for maintaining a proper immune response to infections. Multiple studies have reported the effects of vitamin E treatment on inflammatory cells obtained from animals. *In vitro* studies have demonstrated that supplementation of vitamin E in rats leads to decreased phosphatase activity, reduced superoxide production, and inhibition of PGE2 production in peritoneal macrophages (Sakamoto et al., 1991; Bulger and Maier, 2003). This inhibition of PGE2 is attributed to the suppression of both phospholipase A2 and cyclooxygenase activities in peritoneal macrophages (Sakamoto et al., 1991). *In vitro* studies have shown that enteral supplementation of vitamin E in rats can suppress the production of TNF in both whole blood and peritoneal macrophages when challenged with LPS (Bulger et al., 1997). The activity of lymphocytes seems to be increased in rats treated with vitamin E, as evident from the higher natural killer cell activity and improved response of splenic lymphocytes to Concanavalin A stimulation, and increased phagocytic activity observed in alveolar macrophages of rats (Moriguchi et al., 1990). Vitamin E treatment affects not only macrophages but also other inflammatory cells, including neutrophils and lymphocytes. Neutrophils derived from animals with vitamin E deficiency exhibit elevated levels of membrane LPx, hydrogen peroxide, and impaired chemotaxis and phagocytosis abilities (Zempleni et al., 2013). Similarly, a study has documented a lack of neutrophil phagocytosis in premature infants, and it has been suggested that the administration of vitamin E could potentially expedite the restoration of normal phagocytic activity during the neonatal period (Chirico et al., 1983). Vitamin E deficiency diets have been found to impact lymphocytes, leading to reduced mitogenic responses in both T and B lymphocytes, decreased mixed lymphocyte response, and diminished production of IL-2 (Bendich, 1990).

In an *in vitro* study of mouse peritoneal macrophages co-treated with LPS and vitamin E derivatives, a reduction in the levels of nitric oxide (NO) and prostaglandin E2 (PGE2) was observed compared to the cells subjected to LPS treatment. The application of vitamin E and its derivatives resulted in a notable augmentation of anti-inflammatory properties, as evidenced by a significant decrease in the levels of TNF-α, IFN-γ, IL-1β, and IL-6, in comparison to samples that were solely subjected to LPS treatment (Ng and Ko, 2012). The utilization of tocotrienol-rich fraction as a potential therapeutic agent in the prophylaxis of chronic inflammatory diseases exhibits promising outcomes, primarily attributed to its ability to impede the induction of cyclooxygenase-2 (COX-2) and inducible nitric oxide synthase (iNOS) expression by LPS and also as it exerts a more pronounced inhibitory effect on the expression of nuclear factor kappa B (NF-κB) (Ng and Ko, 2012).

Similarly, the effects of different forms of vitamin E (MitoVitE, α- tocopherol and Trolox) on human umbilical vein endothelial cells (HUVECs) subjected to LPS treatment emulating the conditions associated with sepsis were assessed (Minter et al., 2020). The exposure to LPS resulted in a notable elevation in free radical generation while upon subjecting the cells to treatment with different forms of vitamin E, a discernible decrease in OS, NFκB activation, and interleukin (IL) secretion was observed (Minter et al., 2020). MitoVitE demonstrates the capacity to effectively scavenge radicals (**Figure 3**) at their origin and mitigate mitochondrial uncoupling, as evidenced by its ability to sustain membrane potential and also MitoVitE results in a reduction of NFκB nuclear translocation (Hughes et al., 2005). The downregulation of inducible prostaglandin endoperoxide synthase 2 (PTGS2 or COX-2), a protein implicated in prostanoid biosynthesis and serving as a mediator of the inflammatory response, was observed upon treatment with MitoVitE. Additionally, MitoVitE treatment resulted downregulation of the Toll-like receptor (TLR) signaling cascade and the suppression of various key components for NFκB downstream signalling, namely IκBKB (inhibitor of nuclear factor kappa B kinase subunit beta), NFκB1, MyD88 (myeloid differentiation primary response gene 88), and TRAF6 (TNF receptor-associated factor 6) (Minter et al., 2020). The administration of MitoVitE resulted in the downregulation of STAT1 (involved in both interferon signaling and cell survival) and STAT3 mRNA expression, which have been observed to exhibit upregulation in mononuclear cells derived from sepsis patients (Severino et al., 2014).

Subsequent to the administration of LPS/peptidoglycan (PepG) to elicit sepsis, when rats were subjected to the administration of MitoVitE (Lowes et al., 2013; Thompson et al., 2022), a mortality rate of 25% was observed among the cohort of rats exclusively administered LPS/PepG while, no instances of mortality were recorded within the groups subjected to the MitoVitE- treatment regimen. The adenosine triphosphate to oxygen (ATP:O) ratios in liver mitochondria of rats subjected to LPS and PepG treatment were found to be lower compared to the control group. Conversely, mice treated with MitoVitE exhibited ATP:O ratios that were comparable to those observed in the control group (Lowes et al., 2013; Thompson et al., 2022). Elevated levels of liver damage indicators, specifically alanine aminotransferase (ALT) and aspartate aminotransferase (AST) activity, as well as renal damage indicated by increased creatinine levels, were observed in rats subjected to LPS/PepG treatment, in comparison to the control group. Conversely, rats treated with MitoVitE exhibited reduced ALT and AST activity, along with decreased creatinine levels, when compared to the rats subjected to LPS/PepG treatment. Notably, the levels of oxidative damage (plasma lipid hydroperoxides) were found to be diminished upon administration of MitoVitE, thereby highlighting the potential of vitamin E and its derivatives in mitigating OS, tissue injury, inflammatory responses, and impairments in mitochondrial function within *in vivo* experimental models (Lowes et al., 2013; Thompson et al., 2022). The administration of α-tocopherol to mice prior to the injection of LPS resulted in a notable reduction in the concentration of LPx and IL-6 in the brain homogenates and in the plasma, as compared to the mice that were solely subjected to LPS injection (Godbout et al., 2004). In a dose-dependent manner, the administration of LPS resulted in an elevation of intracellular peroxides and IL-6 levels in primary microglia. However, these effects were mitigated by prior treatment with α-tocopherol (Godbout et al., 2004).

Numerous animal studies and have shown enhanced survival in animal models of sepsis following treatment with α-tocopherol (Marubayashi et al., 1986; Sugino et al., 1987; Powell et al., 1991). Administering α-tocopherol to animal models of sepsis results in a reduction in hepatic LPx, alleviation of disseminated intravascular coagulation, and decrease in plasma lactate levels (Yoshikawa et al., 1984; Bulger and Maier, 2003). α-Tocopherol has demonstrated beneficial effects in various models of excessive inflammation, such as murine renal and hepatic ischemia-reperfusion, and pulmonary inflammation after zymosan-induced peritonitis in rats (Takenaka et al., 1981; Marubayashi et al., 1986; Demling et al., 1995). In the study on liver ischemia-reperfusion, the group treated with α-tocopherol showed reduced LPx level, improved ATP generation, increased survival, and reduced hepatic damage (Marubayashi et al., 1986). In a renal warm ischemia model, pretreatment with α-tocopherol demonstrated kidney protection by increasing ATP levels during reperfusion and reducing serum creatinine levels. The ischemic rats showed improved survival rates following treatment with α-tocopherol (Takenaka et al., 1981). In acute zymosan-induced peritonitis, the prompt administration of α-tocopherol after i.p. zymosan injection resulted in reduced production of pulmonary LPx level and mitigated pulmonary tissue damage compared to the control group (Demling et al., 1995). In another study, the co-administration of selenium and vitamin E exhibited a synergistic effect in rats with sepsis induced by CLP technique, leading to the most favorable outcome in terms of decreasing induced CRP (inflammation marker) levels, enhancing blood oxygenation, and ameliorating lung tissue damage in rats (Atli et al., 2012). The correlation between plasma concentrations of 15-keto-13,14-dihydro-PGF2α (15-K-DH-PGF2α, a cyclooxygenase catalyzed LPx product indicating inflammatory reaction) and 8-iso-prostaglandin F2α (8-iso-PGF2α, a non-enzymatic free radical-mediated LPx product indicating oxidative injury) with the survival outcomes in porcine endotoxemia has been reported (Basu and Eriksson, 2000a). The progressive depletion of vitamin E in the circulatory system, accompanied by an increase in plasma isoprostanes and prostaglandins, disrupts the oxidant-antioxidant balance, potentially impacting survivability during the experimental porcine septicemia (Basu and Eriksson, 2000b).

Sepsis has been linked to decreased levels of vitamin E, which has been shown in various clinical studies to induce accelerated apoptosis, tissue damage, eventual multiorgan system failure, and increased mortality rates (Weber et al., 2008; Dang et al., 2021). The administration of vitamin E to patients with clinical sepsis, as well as *in vivo* models, has demonstrated a decrease in tissue damage and organ failure, accompanied by a reduction in OS (Nathens et al., 2002; Atli et al., 2012). Multiple studies on critically ill patients, including patients in the ICU with septic shock or with significant burns or traumatic injuries, have provided evidence of continuous systemic LPx with elevated levels of plasma TBARS and conjugated dienes and loss of plasma antioxidants including vitamin E (Nguyen et al., 1993; Goode et al., 1995; Bulger and Maier, 2003). Research on patients with acute respiratory distress syndrome (ARDS) has shown an elevation in oxidant activity in bronchoalveolar lavage fluid (Cross et al., 1990) and a significant decrease in vitamin E plasma levels (Richard et al., 1990; Bulger and Maier, 2003). The prevalence of vitamin E deficiency is notably high among pediatric patients who are critically ill and diagnosed with sepsis, thereby playing a contributory role in the development of septic shock. A significant negative correlation was observed between levels of vitamin E and the likelihood of mortality and cardiovascular sequential organ failure assessment scores in critically ill children with infection (Dang et al., 2021).

In a randomized, prospective study, a notable reduction in the incidence of pneumonia and ARDS was observed among patients who were administered α-tocopherol and ascorbic acid, as compared to those who received conventional standard care (Nathens et al., 2002). Furthermore, individuals who underwent α-tocopherol and ascorbic acid treatment exhibited a notable reduction in inflammatory response in alveoli evidenced by a significant decrease in the concentration of white blood cell proteins within the alveoli, as well as lower levels of alveolar fluid levels of F2α isoprostane and TNF-α, IL-1β, and IL-6 concentrations (Nathens et al., 2002). In another clinical trial (Aisa-Alvarez et al., 2020) investigating the impact of various antioxidant interventions on individuals suffering from septic shock and experiencing multiple organ failure showed that the application of vitamin E in patients did not change the SOFA score (a comprehensive assessment of organ dysfunction encompassing neurologic, respiratory, hemodynamic, hepatic, and hematologic aspects) but showed statistically insignificant reduction in LPx (a marker for severity of kidney damage; (Ratliff et al., 2016)) as well as carbonylation contents (Aisa-Alvarez et al., 2020). Nevertheless, the administration of vitamin E resulted in a decrease in procalcitonin (PCT) levels (Aisa-Alvarez et al., 2020). It is important to note that PCT represents a peptide precursor of calcitonin, with its principal stimulus being infection, and is recognized as a component of the complex pro-inflammatory response of the innate immune system (Shiferaw, 2016). The utilization of PCT as a biochemical marker has garnered considerable attention due to its potential efficacy in distinguishing sepsis from various non-infectious etiologies, and it has a higher predictive value for mortality (Paudel et al., 2020).

### 6.3 N-acetylcysteine (NAC)

NAC has garnered considerable attention as a prospective contender due to its cellular protective capabilities and antioxidant attributes in ARDS management and its noteworthy therapeutic efficacy in the treatment of septic ailments (**Table 2** and **Figure 5**) (Ortolani et al., 2000; Rank et al., 2000; Zhang et al., 2017). It exhibits scavenging activity against ROS and also possesses inhibitory effects on pro-inflammatory cytokines (Hsu et al., 2006). The administration of NAC following LPS treatment exhibited a mitigating effect on the reduction in mean arterial pressure while concurrently inducing an elevation in heart rate. Additionally, NAC treatment resulted in a reduction in various indicators of organ injury, namely blood urea nitrogen, glutamic oxaloacetic transaminase, lactate dehydrogenase, creatinine, creatine phosphokinase, and glutamic pyruvic transaminase, demonstrating advantageous effects of NAC, thus conferring protection against LPS-induced renal, cardiac, and hepatic injury in rats (Hsu et al., 2006). Additionally, the administration of NAC subsequent to treatment has been observed to effectively inhibit the secretion of plasma TNF-α, IL-6, and IL-10 in cases of endotoxin shock (Hsu et al., 2006). In another study, administration of NAC prior to endotoxin exposure exhibited a dose-dependent reduction in the activation of nuclear factor kappa B (NF-κB) in the lungs and thus playing an important role in controlling the pulmonary inflammatory response. Furthermore, NAC treatment led to a decrease in the expression of mRNA encoding cytokine-induced neutrophil chemoattractant in the lung tissue and exhibited a notable inhibitory effect on the development of neutrophilic alveolitis induced by endotoxin (Blackwell et al., 1996).

On the contrary, a study elucidating the primary adverse effects associated with NAC treatment shows NAC treatment predominantly manifests as mild gastrointestinal symptoms (Dodd et al., 2008). Undoubtedly, the ramifications of NAC therapy primarily manifest in a benign manner, with any more severe consequences being contingent upon the dosage administered. In the event that the dosage of NAC administered intravenously exceeds 3 grams per day, it is important to note that anaphylactoid reactions may manifest. These reactions encompass a range of symptoms, such as flushing, urticaria, bronchospasm, hypotension, and angioedema (Peake et al., 1996). The use of NAC as an adjunctive treatment in individuals newly diagnosed with septic shock was found to be correlated with a decline in cardiovascular function. This was evidenced by the gradual decrease in cardiac index, left ventricular stroke work index, and mean arterial pressure (Peake et al., 1996). In a prospective, randomized, placebo-controlled study, the early administration of NAC in clinical sepsis has been found to have no effect on microalbuminuria/creatinine ratio, and thus had no effect in reducing endothelial damage. In fact, it has been observed that early administration of NAC can exacerbate endothelial damage, ultimately resulting in sepsis-induced organ failure, particularly cardiovascular failure (Spapen et al., 2005).

### 6.4 Selenium

Selenium exhibits the highest efficacy as an antioxidant agent in clinical conditions (Heyland et al., 2005; Toro-Pérez and Rodrigo, 2021). The mitigation of OS in sepsis can be achieved using antioxidants and selenium that are specifically targeted towards the mitochondria (**Table 2** and **Figures 3** and **5**). Selenium supplementation in animal trials has been found to decrease OS, intranuclear nuclear factor-κB translocation, cytokine formation, and tissue damage (Beck et al., 2001) and restore normal functioning of selenoenzymes, including intracellular GPx and thioredoxin reductase activities. These effects resulted in decreased production of inflammatory prostaglandins and leukotrienes and regulation of the respiratory burst (Rayman, 2000). Selenium is required for the proper operation of GPx and other endogenous antioxidant enzymes. Reduced oxidative equilibrium in the body has been linked to low selenium levels (Barchielli et al., 2022). Selenium deprivation upregulated iNOS expression in macrophages, which in turn upregulated NF-kB production and, ultimately, oxidative damage throughout the body. In LPS-stimulated RAW 264.7 macrophage cell lines, iNOS expression is inversely proportional to cellular selenium levels (Prabhu et al., 2002). The attenuation of LPS-induced ROS and NO production by selenium in murine macrophage cultures was achieved by reducing the expression of inducible NO synthase (iNOS) (Kim et al., 2004). Selenium was found to have preventive effects on inducible nitric oxide synthase (iNOS), which were dependent on the activation of p38 mitogen-activated protein kinase and nuclear factor-kappaB (Huang et al., 2004; Kim et al., 2004).

The antioxidant activity of selenium is observed in its role as a component of various GPx selenoenzymes and other selenoproteins (Burk and Hill, 2005). Multiple studies have consistently demonstrated a reduction in plasma selenium concentration in patients with systemic inflammatory response syndrome (SIRS) and sepsis (Forceville et al., 1998; Angstwurm et al., 1999). The decline in plasma GPx activity exhibits a parallel trend with plasma selenium levels, whereas the administration of selenium supplements reinstates the enzymatic activity (Angstwurm et al., 1999) and demonstrates a correlation with improved health outcomes. A randomized controlled trial involving patients who were administered selenium supplementation appeared to enhance the clinical outcomes and decrease the occurrence of hemodialysis-requiring acute renal failure (Angstwurm et al., 1999).

Another placebo-controlled, multicenter prospective randomized study demonstrates that high-dose selenium supplementation used as an adjuvant treatment reduced mortality rates in patients with sepsis, particularly those with septic shock (Angstwurm et al., 2007). The study reported that patients in the selenium group had a significantly lower 28-day mortality rate of 42.4% compared to 56.7% in the placebo group. During selenium treatment, whole blood selenium concentrations and GPx-3 activity were within the upper normal range, while in the placebo group, these levels remained significantly low (Angstwurm et al., 2007). The specific mechanisms underlying the positive effects of selenium supplementation as an adjuvant are not fully understood. Nevertheless, there is compelling evidence suggesting that selenium may enhance the functions of crucial selenoenzymes responsible for maintaining redox homeostasis, as well as immune and endothelial cell function (Angstwurm et al., 2007). Moreover, it has been suggested that subfertility and mortality rates are predominantly higher in males than in females in cases of sepsis (Barchielli et al., 2022). The male reproductive system has a greater reliance on selenium compared to the female reproductive system. Trials investigating selenium supplementation in sepsis patients had a limited representation of women (Barchielli et al., 2022). The administration of parenteral selenium, either as a standalone treatment or in conjunction with other antioxidants, has been found to be both safe and potentially linked to a decrease in mortality rates among critically ill individuals (Heyland et al., 2005). Nevertheless, it is imperative to conduct additional research to thoroughly examine the effectiveness and safety of selenium. This is particularly important as the consumption of selenium at a dosage exceeding 0.4 mg/day in adult individuals has been associated with the potential manifestation of unfavorable consequences over an extended period of time (Kim et al., 2004).

### 6.5 Glutathione (GSH)

Glutathione (GSH) is recognized as a prominent scavenger, supported by increasing evidence that links the reduction of GSH levels to the severity of various pathological conditions (**Table 2**). The importance of GSH and other antioxidants in preventing LPx that would otherwise follow from the unchecked spread of free-radical reactions previously reported (Sahoo et al., 2005; Sahoo et al.,2006; Chattopadhyay et al., 2007; Sahoo et al., 2007; Sahoo and Chainy, 2007; Sahoo et al., 2008a; Sahoo et al., 2008b; Sahoo et al., 2008c; Chattopadhyay et al., 2010; Sahoo, 2012; Sahoo and Roy, 2012; Sahoo and Roy, 2012; Sahoo et al., 2019; Sahoo et al., 2019; Chainy and Sahoo, 2020; Sahoo et al., 2022; Sahoo and Chainy, 2023; Sahoo et al., 2023a; Sahoo et al., 2023b). Supplementing glutamine (a precursor of glutathione) (Amores-Sánchez and Medina, 1999) in enteral and parenteral nutrition solutions can help maintain elevated levels of GSH and protect against OS damage (Wernerman and Hammarqvist, 1999). Several trials conducted in ICU patients demonstrated the effectiveness of glutamine-supplemented parenteral nutrition in preventing infections (Houdijk et al., 1999; Goeters et al., 2002; Griffiths et al., 2002). Another multicenter trial involving 114 critically ill patients demonstrated that the use of alanine- and glutamine-supplemented parenteral nutrition resulted in a lower incidence of infectious complications and improved metabolic tolerance (Déchelotte et al., 2006; Su et al., 2021).

Consistent with other research (Koksal et al., 2004; Şener et al., 2005), CLP was found to reduce tissue GSH levels, which may be attributable to its consumption in sepsis-induced OS. Septic animals exhibit an elevated turnover of GSH, indicating an increased utilization of this antioxidant (Malmezat et al., 2000). The rates of GSH synthesis were significantly higher in the lung, muscle, spleen, liver, large intestine, and heart of infected rats. The liver of infected rats exhibited significantly higher activities of liver gamma-glutamyl-cysteine synthetase and glutathione reductase (GR), which are involved in the stimulation of liver glutathione synthesis (Malmezat et al., 2000). Consequently, the potential to manipulate glutathione availability has become an appealing approach for sepsis treatment (Wernerman and Hammarqvist, 1999).

### 6.6 Carnosine

Carnosine is a dipeptide endogenously synthesized from β-alanine, a non-proteogenic amino acid, and L-histidine, an essential amino acid. Carnosine is found in the human body in relatively high concentrations in excitable tissues (brain and skeletal muscle) but also in lower quantities in other tissues (kidney, gastrointestinal tract, adipose tissue, heart, and liver) (Cesak et al., 2023). Due to its several modes of action, carnosine supplementation has promise as an additional therapy for the treatment of sepsis (Bonaccorso et al., 2023). Carnosine’s antioxidant qualities, anti-inflammatory actions, and capacity to boost cellular energy generation may help alleviate the systemic inflammation, OS, and mitochondrial dysfunction that characterize sepsis (Caruso et al., 2023). Carnosine has been shown to have antioxidant effects, and lower concentrations of carnosine result in increased levels of OS (Su et al., 2015). Carnosine has also been demonstrated to regulate immunological responses and prevent the production of advanced glycation end products, which can aggravate organ dysfunction brought on by sepsis (Byun et al., 2017). According to preliminary research, supplementing with carnosine may help septic patients recover more quickly and have better results by lowering the severity of organ damage (Jindal et al., 2020).

An investigation into the therapeutic efficacy of carnosine has yielded promising results, as evidenced by a notable reduction in the severity of sepsis, as indicated by histopathological analysis and carnosine has demonstrated a favorable effect by renormalizing LPx and antioxidant enzymes and lowering levels of IL-β, TNF-α, and macrophage inhibitory factor (Sun et al., 2017; Jukić et al., 2021). In several animal studies, it has been observed that carnosine exhibits the potential to mitigate acute renal injury induced by septic shock. Rats treated with carnosine showed notable enhancements in kidney function, tissue and serum malondialdehyde (MDA) levels, biochemical indices, routine blood values, and histopathological findings in comparison to rats exposed to septic shock without carnosine treatment (Sahin and Donmez, 2018). In another study on the murine septic shock model, significant improvements were found in liver function, serum and tissue MDA levels, and histological findings when treated with carnosine, compared to rats with untreated sepsis. In comparison to the sepsis group, rats treated with carnosine experienced less sinusoidal dilatation and cellular inflammation in the portal region; the livers of rats in this group showed near-normal histological structure (Sahin et al., 2013). Similarly, a study investigating the impact of carnosine on an ARDS murine model demonstrated that carnosine effectively reduced vascular permeability, tissue damage, and lung inflammation induced by LPS. The administration of carnosine mitigated the increase in ROS levels induced by LPS administration. The inhibitory effects of carnosine on neutrophilic inflammation induced by LPS were observed through the reduction in MPO activation in the lung and the decrease in extracellular DNA levels in bronchoalveolar lavage fluid. Furthermore, the administration of carnosine exhibited a suppressive effect on the *in vivo* activation of the endoplasmic reticulum (ER) stress response that was induced by LPS (Tanaka et al., 2017).

### 6.7 Fish oil/Omega-3 fatty acid supplementation

Fish oil, which contains the omega-3 fatty acids eicosapentaenoic acid (EPA) and docosahexaenoic acid (DHA), has been studied for its possible use in the treatment of sepsis due to its anti-inflammatory effects (**Table 2**) (Rogero et al., 2020). It has been shown that omega-3 fatty acids can modify the immune response, lessen inflammation, and enhance vascular function—all of which are important for treatment of sepsis (Gutiérrez et al., 2019). Omega-3 supplements can potentially improve outcomes by reducing the overwhelming inflammatory response found in sepsis (Lu et al., 2017).

The interplay between nutritional intervention, lipid profile, and survival yields intriguing connections, with discernible alterations in lipid metabolism being evident among individuals in critical conditions (Green et al., 2016). Through the inhibition of pro-inflammatory (eicosanoid, NF-kB) and the promotion of anti-inflammatory (resolvin, protectin) mediators, omega-3 fatty acid nutritional supplementation has been proposed to modify the immune response in critical disease (Zhao et al., 2021). Clinical evidence for the possible advantages of omega-3 fatty acids in ARDS and general critical illness has been moderated by research revealing ambiguous effects and even potential damage (Koekkoek et al., 2019).

A lower ratio of arachidonic acid to eicosapentaenoic acid and docosahexaenoic acid following treatment with omega-3 fatty acids may be linked to a better prognosis for critically ill sepsis patients (Hall et al., 2016). A recent meta-analysis of twelve randomized clinical trials (RCTs) (721 patients) investigated the effects of parenteral omega-3 in sepsis. Their findings demonstrated a substantial decrease in 28-day mortality and ICU length of stay (LOS) but no effect on duration of mechanical ventilation (DMV) (Yanping et al., 2014). Supplementation with omega-3 fatty acids may lower mortality in sepsis patients, particularly those with gastrointestinal dysfunction. Furthermore, omega-3 fatty acid supplementation may reduce DMV and ICU LOS (Wang et al., 2020). Omega-3 fatty acid supplementation can lower the mortality rate of sepsis and sepsis-induced acute respiratory distress syndrome (Chen et al., 2018). According to a meta-analysis (Yanping et al., 2014), patients with sepsis who received parenteral nutrition (PN) with fish oil supplementation had lower fatality rates, shorter stays in ICUs, and shorter hospital stays. Similarly, Wei et al. (2010) also reported that patients receiving treatment with a lipid emulsion, including fish oil, had considerably shorter hospital stays and fewer infection problems (Wei et al., 2010).

The use of fish oil (rich in omega-3 fatty acids) has demonstrated a potential protective effect against organic dysfunction caused by sepsis, evidenced by the observed decrease in lactate levels in the serum of septic Wistar rats and mitigating liver injury by lowering ALT levels (Velasque et al., 2023). Omega-3 fatty acid treatment has also led to a decrease in ROS and OS, resulting in reduced levels of TBARS and DCF (2’,7’-dichlorofluorescein, a fluorescent product for detecting ROS) in serum and liver tissue. Also, the sepsis group having omega-3 fatty acids exhibited increased activity of CAT and GPx in liver tissue. Additionally, this group showed higher levels of thiols in serum compared to the sepsis group. Levels of creatinine and urea were decreased as a result of omega-3 fatty acids treatment (Velasque et al., 2023). The encouraging outcomes demonstrate that omega-3 fatty acids, which function as an anti-inflammatory and potent antioxidant, are a potentially effective treatment for liver injuries and alterations caused by sepsis, particularly in OS conditions (**Table 2** and **Figure 5**) (Velasque et al., 2023).

### 6.8 Antioxidant multitherapy

Numerous studies have already substantiated the efficacy of antioxidant therapy in attenuating inflammatory responses, thereby mitigating the pathogenesis of sepsis. Considering the limited efficacy observed in clinical trials regarding the utilization of antioxidants as a standalone treatment modality for sepsis, it is recommended to explore the implementation of a multifaceted therapeutic approach. The utilization of therapeutic interventions that exhibit additive or synergistic effects on multiple pathways involved in bolstering the antioxidant defense mechanism is a subject of considerable interest. This can be achieved through the administration of a diverse array of antioxidant agents.

The amelioration of inflammation in individuals diagnosed with septic shock is accomplished through the supplementation of various antioxidants to the conventional therapeutic regimen (Aisa-Alvarez et al., 2020). In pulmonary sepsis, a multifaceted therapeutic approach using antioxidants vitamin C, NAC, and vitamin E has been reported. In pulmonary sepsis, it has been observed that the implementation of vitamin C replacement therapy leads to a reduction in the levels of C-reactive protein (CRP), procalcitonin (PCT), as well as nitrate/nitrite (NO3−/NO2−). Furthermore, the administration of N-acetylcysteine (NAC) has demonstrated the ability to decrease LPx and enhance the antioxidant capacity while the utilization of vitamin E has shown a tendency to diminish LPx. It can be posited that every individual antioxidant made a notable contribution towards yielding a favorable outcome (Aisa-Alvarez et al., 2020).

In another study, following the induction of sepsis in rats, a therapeutic intervention involving the administration of a combination of ceftriaxone, along with NAC and vitamins C and E, was implemented. The survival rate in the group receiving ceftriaxone plus antioxidants was observed to be higher than the control group, which solely received ceftriaxone (Galvão et al., 2014). Moreover, this study revealed that the introduction of antioxidants exhibited a significant correlation with a reduction in the levels of superoxide radical anion, LPx, and PC contents (Galvão et al., 2014), all of which serve as indicative indicators of OS.

## 7 Importance of immunoadjuvant therapy

IL-7, a cytokine, exhibits a plethora of supplementary actions that manifest considerable advantageous effects in sepsis, and it has the capacity to counteract a significant pathological aberration observed in sepsis. For example, the administration of IL-7 has been observed to inhibit the process of lymphocyte apoptotic cell death that is induced by sepsis. This effect is achieved through the upregulation of the anti-apoptotic molecule Bcl-2 (Unsinger et al., 2010; Venet et al., 2012). Furthermore, IL-7 enhances the proliferation of lymphocytes by exerting its influence on the activation of the PI3 kinase pathway (Venet et al., 2012). The intervention of IL-7 holds promise for the restoration of functional capabilities in T cells that are characterized by hyporesponsiveness or exhaustion, a common feature observed in sepsis (Kasten et al., 2010; Unsinger et al., 2010, 2012; Venet et al., 2012). IL-7 facilitates communication between Th1 and Th17 lymphocytes in sepsis, resulting in enhanced neutrophil recruitment and bacterial clearance without increasing early tissue injury (Kasten et al., 2010). The administration of IL-7 exhibited a beneficial effect on the decline of immune effector cells, thereby enhancing various lymphocyte functions such as activation, proliferation, expression of adhesion molecules, and production of interferon-γ. The observed advantageous impacts of IL-7 were found to be correlated with an augmentation in overall immune function, as evidenced by an intensified delayed-type hypersensitivity reaction and a notable enhancement in survival rates (Unsinger et al., 2012). Furthermore, the findings of a preclinical study examining the efficacy of recombinant human IL-7 (rhIL-7) as a lymphostimulating intervention in sepsis revealed that the ex vivo administration of rhIL-7 to patients’ cells resulted in a noteworthy enhancement of lymphocyte functionality in various aspects, including the proliferation of CD4(+) and CD8(+) lymphocytes, the production of IFN-γ, the phosphorylation of STAT5, and the induction of B cell lymphoma 2 following stimulation (Venet et al., 2012). Furthermore, it has been observed that this increase in expression also leads to a greater diversity in T-cell receptors. Consequently, this enhanced diversity contributes to a more robust and potent immune response against various pathogens (Lévy et al., 2012; Morre and Beq, 2012). The reversal of T-cell dysfunction and subsequent pathogen clearance can be achieved through the utilization of antibody blockade targeting PD-1 or its ligand, PD-L1. The inhibition of the PD-1 pathway results in enhanced survival outcomes in animal models of bacterial and fungal sepsis that closely resemble the clinical context (Brahmamdam et al., 2010; Zhang et al., 2010).

IL-15, a versatile cytokine, exhibits a close relationship with IL-7 (Inoue et al., 2010). It exerts its effects on both CD4 and CD8 T cells, stimulating their proliferation while concurrently inhibiting apoptosis. One potential advantage of IL-15 in comparison to IL-7 lies in its robust immunostimulatory and proliferative properties, particularly regarding natural killer cells and dendritic cells. The cellular entities play crucial roles in the immune response against pathogenic invasion while concurrently experiencing significant depletion during pathological conditions like sepsis. The administration of IL-15 exhibited a significant inhibitory effect on sepsis-induced apoptosis of CD8 T cells, natural killer cells, and dendritic cells. Furthermore, this intervention demonstrated a notable enhancement in survival rates among subjects afflicted with sepsis resulting from CLP, as well as *P. aeruginosa* pneumonia (Inoue et al., 2010). Similarly, the administration of interferon γ (IFN-γ) exhibited a notable reversal of the monocyte dysfunction induced by sepsis, leading to a successful resolution of the septic insult (Nalos et al., 2012). The IFN-γ therapy in persistent staphylococcal sepsis elicited a notable upregulation of human leukocyte antigen complex (HLA-DR) expression on monocytes, accompanied by a discernible augmentation in the population of CD4 T cells producing IL-17. Furthermore, this therapeutic intervention demonstrated a favorable clinical outcome, leading to the resolution of sepsis (Nalos et al., 2012).

The efficacy of an immunostimulatory therapeutic strategy hinges upon the implementation of personalized, precise, and temporally optimized interventions. It is imperative to note that the aforementioned approach exclusively confers advantages to septic patients who exhibit immunosuppressive tendencies. The expeditious identification of sepsis-induced epigenetic alterations in specific patients afflicted with sepsis has the potential to facilitate prompt recognition of an immunosuppressive condition, thereby enabling the administration of immune-enhancing therapy in a more timely manner.

## 8 Conclusion

In summary, while the primary therapeutic approach for sepsis typically revolves around fluid therapy and antimicrobial agents, it is worth considering the potential role of antioxidants as an adjunct in the management of this medical condition. While antioxidants with simultaneous antimicrobial capabilities may appear promising as an adjunct therapy for sepsis, it is important to carefully evaluate the pros and cons of using antioxidants in the therapeutic approach to sepsis. In addition, the precise timing and the specific clinical circumstances of the individual must be taken into careful consideration when determining the appropriate administration of antioxidants. Antioxidant multitherapy is recommended alongside the primary treatment for sepsis, considering the lack of substantial benefit observed in clinical trials involving antioxidant monotherapy. The administration of a variety of antioxidant agents presents a favorable approach in therapeutic interventions, as it allows for the utilization of treatments that are enhanced by the additive or synergistic interactions across multiple pathways, thereby augmenting the overall antioxidant defense mechanisms (Marshall, 2014). Immunoadjuvant therapy using IL-7 and IL-15, administration of interferon γ, and targeting PD-1, particularly to septic patients who exhibit immunosuppressive tendencies, signifies a significant forthcoming breakthrough in the realm of sepsis. However, in instances characterized by the occurrence of a cytokine storm, wherein an exacerbated inflammatory reaction is observed, such as in the case of ARDS, the administration of antioxidants has been postulated to yield potential benefits in the suppression of the immune response. It is noteworthy to mention that the implementation of research-based recommendations regarding the acute management of sepsis and septic shock is crucial for improving outcomes for critically ill patients with excessive mortality rates (Rhodes et al., 2017). In order to establish reliable recommendations, it is crucial to conduct thorough research, particularly through well-designed randomized controlled trials that explore various aspects of multi-antioxidant therapy or immunoadjuvant therapy.

## Author contributions

DS: Conceptualization, Data curation, Formal Analysis, Investigation, Methodology, Resources, Software, Visualization, Writing – original draft, Writing – review & editing. DW: Conceptualization, Funding acquisition, Writing – review & editing. AP: Investigation, Writing – review & editing. BP: Data curation, Writing – review & editing. VY: Investigation, Writing – review & editing. AsP: Conceptualization, Supervision, Writing – review & editing. AJ: Conceptualization, Writing – review & editing.
